# Metagenomic Insights Into the Changes of Antibiotic Resistance and Pathogenicity Factor Pools Upon Thermophilic Composting of Human Excreta

**DOI:** 10.3389/fmicb.2022.826071

**Published:** 2022-03-31

**Authors:** Katharina A. Werner, Dominik Schneider, Anja Poehlein, Nina Diederich, Lara Feyen, Katharina Axtmann, Tobias Hübner, Nicolas Brüggemann, Katharina Prost, Rolf Daniel, Elisabeth Grohmann

**Affiliations:** ^1^Department of Microbiology, Faculty of Life Sciences and Technology, Berliner Hochschule für Technik, Berlin, Germany; ^2^Department of Genomic and Applied Microbiology and Göttingen Genomics Laboratory, Institute of Microbiology and Genetics, Georg-August-University Göttingen, Göttingen, Germany; ^3^Institute for Medical Microbiology, Immunology and Parasitology, University Hospital Bonn, Bonn, Germany; ^4^Department of Environmental Microbiology, Helmholtz Centre for Environmental Research GmbH—Umweltforschungszentrum Leipzig (UFZ), Leipzig, Germany; ^5^Institute of Bio- and Geosciences—Agrosphere (IBG-3), Forschungszentrum Jülich, Jülich, Germany

**Keywords:** compost, ecological sanitation, human excreta, bacterial community, antibiotic resistance, pathogenicity, metagenomics, qPCR (quantitative PCR)

## Abstract

In times of climate change, practicing a form of sustainable, climate-resilient and productive agriculture is of primordial importance. Compost could be one form of sustainable fertilizer, which is increasing humus, water holding capacity, and nutrient contents of soils. It could thereby strengthen agriculture toward the adverse effects of climate change, especially when additionally combined with biochar. To get access to sufficient amounts of suitable materials for composting, resources, which are currently treated as waste, such as human excreta, could be a promising option. However, the safety of the produced compost regarding human pathogens, pharmaceuticals (like antibiotics) and related resistance genes must be considered. In this context, we have investigated the effect of 140- and 154-days of thermophilic composting on the hygienization of human excreta and saw dust from dry toilets together with straw and green cuttings with and without addition of biochar. Compost samples were taken at the beginning and end of the composting process and metagenomic analysis was conducted to assess the fate of antibiotic resistance genes (ARGs) and pathogenicity factors of the microbial community over composting. Potential ARGs conferring resistance to major classes of antibiotics, such as beta-lactam antibiotics, vancomycin, the MLS_B_ group, aminoglycosides, tetracyclines and quinolones were detected in all samples. However, relative abundance of ARGs decreased from the beginning to the end of composting. This trend was also found for genes encoding type III, type IV, and type VI secretion systems, that are involved in pathogenicity, protein effector transport into eukaryotic cells and horizontal gene transfer between bacteria, respectively. The results suggest that the occurrence of potentially pathogenic microorganisms harboring ARGs declines during thermophilic composting. Nevertheless, ARG levels did not decline below the detection limit of quantitative PCR (qPCR). Thresholds for the usage of compost regarding acceptable resistance gene levels are yet to be evaluated and defined.

## Introduction

Human excreta contain large amounts of key nutrients for agriculture, like nitrogen, phosphorus, and potassium. However, due to sanitation systems that treat human excreta as waste, they usually remain unused in many countries that use water-based sanitation systems. Ecological sanitation using thermophilic composting can serve as a treatment to convert human excreta into a humus-rich material with low pathogen contents that nurtures plants and increases soil fertility of agricultural fields (Dalzell et al., 1987; Amlinger et al., 2007; Rose et al., 2015; Reid, 2020; Ryals et al., 2021). The possible spread of human pathogens or ARGs is one concern toward the usage of composted human excrements (Schönning and Stenström, 2004). The positive effect of compost on soil fertility can be even additionally increased by the use of biochar-compost, i.e., compost containing co-composted charcoal pieces (Fischer and Glaser, 2012; Agegnehu et al., 2017).

Thermophilic composting is the aerobic, exothermic degradation of organic material through biological activity. The process comprises a first mesophilic, a thermophilic, a second mesophilic and a cooling or maturation phase, which are dominated by different microbial species (Bernal et al., 2009). Heat-tolerant bacteria are predominant in the thermophilic phase; fungi and yeasts proliferate when temperatures decrease again in the second mesophilic and maturation phases. Members of the bacterial phyla *Firmicutes* and *Proteobacteria*, followed by *Actinobacteria*, *Bacteroidetes*, or *Planctomycetes* are usually dominant throughout composting, although the composition differs with the composting conditions (Ryckeboer et al., 2003; Esperón et al., 2020). Biochar addition to composting was found to alter the microbial community through altered C:N ratios or reduced amounts of dissolved organic carbon (Jindo et al., 2012; Qiu et al., 2019). Biochar can additionally affect the removal of ARGs, but the effect seems to be dependent on the type of pyrolyzed material used for co-composting (Cui et al., 2016).

Antibiotic resistant bacteria pose a growing risk in the modern world due to limited treatment options. Increased usage of antibiotics enhances the selective pressure leading to the accumulation of multiple resistances in one strain, which has been estimated to cause at least 700,000 deaths per year (Laxminarayan et al., 2013; O’Neill, 2014). Methicillin-resistant *Staphylococcus aureus* (MRSA) or vancomycin-resistant enterococci (VRE) are declared high priority pathogens for research and development of new antibiotics by the WHO (Tacconelli et al., 2018). The level of ARGs in soils has increased with increasing use of antibiotics over the last decades, and transfer of ARGs from environmental to clinical strains has been reported (Lartigue et al., 2006; Walsh, 2006; Knapp et al., 2010; Graham et al., 2016). Hence, it is of great importance to interrupt possible cycles of ARG transfer. The application of untreated manure was shown to increase ARG levels in the field (Knapp et al., 2010); however, thermophilic composting as a pretreatment can decrease the load of ARGs. Lower levels of beta-lactam, macrolide, quinolone, sulfonamide and tetracycline resistance genes through thermophilic composting were reported for manure (Wang et al., 2016; Guo et al., 2017; Chen Z. et al., 2018; Esperón et al., 2020). However, variations in the reduction efficiency, inconclusive results for sewage sludge composting, and limited studies on the fate of ARGs during human waste composting, especially when co-composted together with biochar, call for more research to get better and reliable insights into the ARGs dispersal dynamics (Zhang et al., 2016; Ezzariai et al., 2018; Jang et al., 2018; Liao et al., 2018; Cui et al., 2020; Wei et al., 2020).

Virulence factors have different functions that enable bacteria to infect host cells and cause disease; their presence differentiates pathogenic from non-pathogenic strains. These functions include adherence factors, siderophores, endo- and exotoxins, invasins, as well as type III and IV secretion systems. The corresponding genes are usually located on mobile genetic elements (MGEs) and often organized in pathogenicity islands that have evolved *via* different mechanisms (Dobrindt et al., 2004; Gal-Mor and Finlay, 2006). Genetic material located on MGEs can be transferred between bacteria of the same or different species through horizontal gene transfer. MGEs comprise for example plasmids, transposons, integrons, and bacteriophages which can be transferred *via* conjugation, transformation or transduction (Dobrindt et al., 2004; Gillings, 2014).

To elucidate the effect of thermophilic composting of human excreta (feces and urine) from dry toilets together with green cuttings and straw, with and without biochar addition, we assessed the effect on ARGs and bacterial virulence genes, as well as the composition of the microbial community. Here, we present metagenomic sequencing and analysis of samples before and after 5 months of composting.

## Materials and Methods

### Composting Trial, Sampling, and Sample Pretreatment

The composting trial (including feedstock material properties, composition and temperature measurements), sampling and sample pretreatment were described in detail in Werner et al. (2022). In brief, thermophilic windrow composting of dry toilet contents, green cuttings, straw, and urine, with and without biochar, was conducted in Eckernförde, Germany, between September 2018 and January 2019. The trial comprised four piles and two treatments. Each of the two treatments (with and without biochar) was set up twice at an interval of 2 weeks, with E1 and E1-B referring to the replicates, which were set up first, and E2 and E2-B referring to the replicates set up 2 weeks later. “-B” indicates the biochar treatment.

Sampling was conducted at the day of the experimental set-up (“start” samples), after 2 weeks (only repetition 1) and at the end of the experimental trials after 5.5 (repetition 1) and 5 months (repetition 2), respectively.

### DNA Extraction and Pooling for Metagenomic Sequencing

Extraction of total DNA was conducted from 250 mg compost using the NucleoSpin^®^ Soil gDNA extraction kit (Macherey-Nagel) according to the manufacturer’s instructions. Lysis buffer SL2 was used without enhancer. Mechanical disruption was performed in a FastPrep-24™ 5G homogenizer (MP Biomedicals) at 5 m/s for 30 s. For DNA elution, 50 μL of nuclease-free water were applied.

For metagenomic analysis, triplicate end samples were pooled equimolarly. A total of eight samples (start and end samples of four compost piles) were sequenced at Göttingen Genomics Laboratory, Germany.

For qPCR analysis, the DNA extracts were further purified using a magnetic separation device NucleoMag^®^ SEP (Macherey-Nagel) and CleanNGS kit (CleanNA) according to manufacturer’s instructions. DNA was stored at −20°C.

### Metagenomic Sequencing

Illumina paired-end sequencing libraries were prepared using the Illumina^®^ DNA Prep, (M) Tagmentation Kit and the Nextera™ DNA CD Indexes kit as recommended by the manufacturer (Illumina, San Diego, CA, United States). To assess quality and size of the libraries, samples were run on an Agilent Bioanalyzer 2100 using an Agilent High Sensitivity DNA Kit as recommended by the manufacturer (Agilent Technologies, Waldbronn, Germany). Concentration of the libraries were determined using the Qubit^®^ dsDNA HS Assay Kit as recommended by the manufacturer (Life Technologies GmbH, Darmstadt, Germany). Sequencing was performed by using the HiSeq2500 instrument (Illumina Inc., San Diego, CA, United States) using the HiSeq Rapid PE Cluster Kit v2 for cluster generation and the HiSeq Raid SBS Kit (500 cycles) for sequencing in the paired-end mode and running 2 × 250 cycles.

### Data Availability

The raw reads of the 8 sequenced metagenomic samples have been deposited in the NCBI Sequence Read Archive, study SRP347939, at BioProject PRJNA782422 under accession numbers SRR17041395 (E1 start), SRR17041394 (E1-B start), SRR17041392 (E2 start), SRR17041391 (E2-B start), SRR17041390 (E1 end), SRR17041389 (E1-B end), SRR17041388 (E2 end) and SRR17041387 (E2-B end). Detailed information is stated in Supplementary Table 1.

### Data Analysis

Metagenomic paired-end sequences of 8 samples were quality-filtered with fastp v0.20.0 (Chen S. et al., 2018) using default settings with the addition of an increased per base phred score of 20, base pair corrections by overlap, as well as 5′- and 3′-end read trimming with a sliding window of 4, a mean quality of 20 and a minimum sequence length of 50 bp. After quality filtering, the metagenome sequences consisted of between 44,872,702 and 94,600,400 paired-end reads with an average read length of 236–239 bp (Supplementary Table 2). Taxonomic assignment was performed by a combination of Kraken2 v2.0.8-beta (Wood et al., 2019) and Kaiju v1.7.0 (Menzel et al., 2016) against the nucleotide (nt) or non-redundant protein (nr) database (retrieved on 22 July, 2020), respectively, to improve sequence classification. The Kraken2 classification was favored over Kaiju classification due to higher accuracy of nucleotide-based classification and taxonomy was added by “addTaxonNames” script of Kaiju. Functional classification of reads was performed with KaijuX (Menzel et al., 2016) with default settings against the KEGG database (Kanehisa et al., 2009) (retrieved on 2018-10-01). *P*-values for functional groups as assigned by the KEGG database were calculated using two-tailed, paired Student’s test. Due to the low number of replicates, start and end samples of all four compost piles were compared. *P*-values below 0.05 were considered significant (*), *p* < 0.01 (^**^) highly significant.

Metagenomic assembly was performed with SPAdes v3.14.0 (Bankevich et al., 2012) using defined kmers (-k 21,33,55,77,99,127) which resulted in 8 assembled datasets. Contigs with length < 1,000 bp were discarded for further processing. Open reading frames (ORFs) from all contigs were determined with PRODIGAL v2.6.3 (Hyatt et al., 2010). Subsequently, all protein sequences were combined and dereplicated with USEARCH v9.2.64 (Edgar, 2010). The protein sequences of predicted ORFs were aligned against the Resfams v1.2.2 (Gibson et al., 2015) core genes database using HMMER v3.3^1^ with the recommended parameters to identify ARG-like ORFs which are referred to as ARGs. Relative abundance of ARGs of each sample was calculated by mapping reads with Bowtie2 v2.3.5.1 (Langmead and Salzberg, 2012) against the ARG nucleotide sequences. SAMtools v1.9 (Li et al., 2009) was used to extract mapped read counts to ARG sequences. Contigs carrying ARGs were taxonomically classified with CAT v5.1.2 (von Meijenfeldt et al., 2019) and used to determine ARG taxonomic origin.

Data basis for MaAsLin2 analysis were genes assigned by Prodigal (Hyatt et al., 2010) from assembled metagenomes. ARG annotation was conducted with HMMer against the Resfams database (Gibson et al., 2015) and taxonomic origin was assigned using CAT (von Meijenfeldt et al., 2019) on the contig carrying the ARG.

Data manipulation and visualization was performed with ampvis2 v2.6.5 (Andersen et al., 2018). Bar charts of superkingdoms were generated with ggplot2 v3.3.2 (Wickham, 2016) and read counts were converted to relative abundances. PCoA based on taxonomic composition was performed at same sequencing effort for (44,090,688 reads per sample). Diversity indices (Richness, Shannon) were calculated with ampvis2 and visualized with ggplot2. Normalization was conducted through conversion of read counts to relative abundances. 77–83% could be assigned taxonomically at kingdom level, 53–69% functionally.

### Quantitative Real Time PCR

qPCR analyses were conducted on selected genes conferring resistance to different classes of antibiotics to quantify the gene concentrations in samples before, after 14-days (only repetition 1) and after composting. Genes were chosen based on previous PCR results of the samples (*aph(2″)-Ia*, *aph(3′)-IIIa*, *ermA*, *ermB*, *sul1*, *sul2*, *tetL* and *tetS*) (Werner et al., 2022). qPCR was performed using a LightCycler^®^ 480 II instrument (Roche Diagnostics Ltd.) and the LightCycler^®^ 480 Probes Master (Roche Diagnostics Deutschland GmbH). Preliminary tests of the DNA extracts revealed an optimal dilution of 1:100 of the template DNA to reduce inhibition of the qPCR. Primer and probe sequences, programs and bacterial strains used for the generation of positive controls are shown in Supplementary Table 3 and composition of the qPCR reactions in Supplementary Table 4. A standard curve of 10^1^–10^8^ gene copies was used for quantification in every run. Each sample was measured in triplicates in each run, and the average of three individual runs is presented. qPCR results were normalized to the 16S rRNA gene copy number, as well as to 1 g compost (dry weight). *P*-values were calculated on the normalized target copy numbers using two-tailed, paired Student‘s test. Due to the low number of replicates, start and end samples of all four compost piles were compared. For this reason, the average of the triplicates from the end of composting was used for analysis. *P*-values below 0.05 were considered significant (*), *p* < 0.01 (^**^) highly significant.

## Results

### Thermophilic Composting Changes the Microbial Community Composition

**FIGURE 1:**
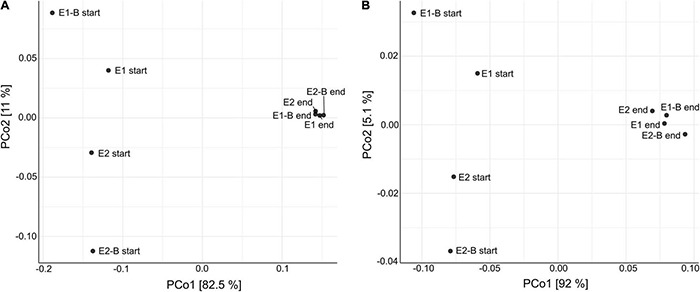
Principal coordinates analysis (PCoA) of the metagenomic data depicting the dissimilarity of each individual sample toward all others. **(A)** Dissimilarity of taxonomic data is depicted in the left diagram with PCo1 (x-axis) explaining 82.5% of differences and PCo2 (y-axis) explaining 11%, respectively. **(B)** Functional data in the right diagram with PCo1 explaining 92% of differences and PCo2 explaining 5.1%, respectively. Each data point represents one of the 8 compost samples of the trial {start and end samples of two treatments [without and with biochar (B)] and two repetitions (E1/E2)}.

**FIGURE 2:**
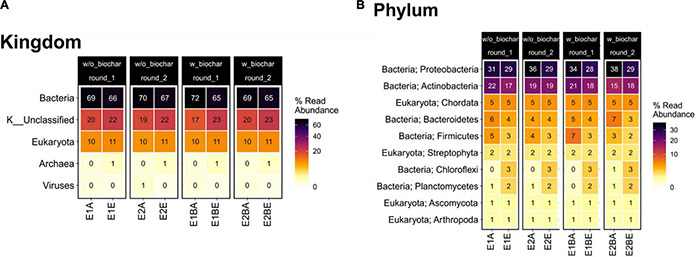
Heatmap depicting relative read abundance on taxonomic kingdom **(A)** and phylum levels **(B)** for the metagenomic data. Samples from start (A) and end (E) of two separate repetitions (1 and 2) of compost treatments without and with biochar (B) are depicted individually.

Composition of the compost community including prokaryotes, eukaryotes and viruses changed from start to end of composting, as suggested by principal coordinates analysis (PCoA). The functional profile of the metagenomic data also exhibits distinct differences, drawing a similar picture as the taxonomic PCoA (Figure 1). Bacteria represented the largest group with around 70% relative abundance in the start and 65–67% in the end samples, followed by Eukaryota that increase from 10 to 11% relative abundance. Archaea and viruses accounted for a maximum of 1% each. At the phylum level, *Proteobacteria* (start: 31–38%; end: 28–29%) and *Actinobacteria* (start: 15–22%; end: 17–19%) showed highest relative abundance in samples from both start and end of the composting. The phyla following in abundance accounted for less than 10% of the metagenomic reads (Figure 2). The composition of the bacterial community was elucidated previously based on 16S rRNA gene amplicon sequencing (Werner et al., 2022). The order of phyla (regarding their relative abundances) differed between amplicon and metagenomic data, but phyla with highest abundance were the same and general trends mostly corresponded. *Proteobacteria* exhibited highest relative abundances in both amplicon and metagenomic sequencing with decreases from the beginning to the end of composting. *Actinobacteria* increased in abundance during composting in the amplicons, whereas averaged metagenomics relative abundance revealed no differences. *Firmicutes* showed the most pronounced decrease in both data sets, but metagenomics detected lower overall abundances. Amplicon and metagenomics both revealed decreases in relative abundance of *Bacteroidetes* during composting. Data on *Chloroflexi* was very similar with strong, almost sixfold increases of the initial relative abundances. In both cases, *Chloroflexi* rose from the fifth to the fourth most abundant phylum during composting (Supplementary Figure 1).

**FIGURE 3:**
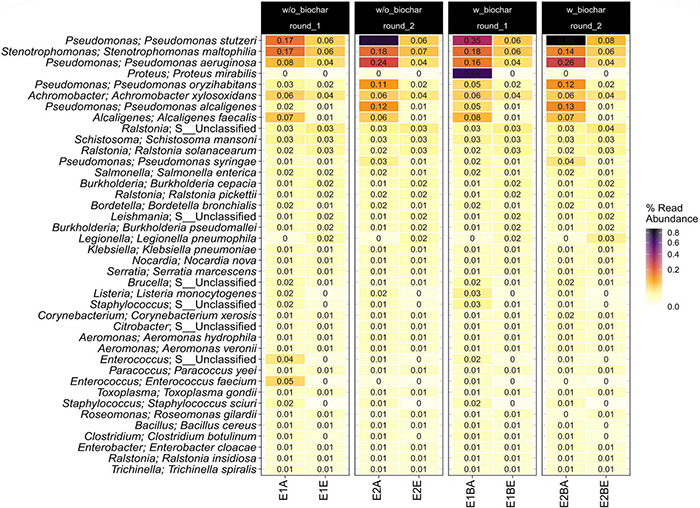
Heatmap showing the 40 most abundant potential pathogenic species and genera in the metagenomic data set as relative read abundance. Samples from start (A) and end (E) of two separate repetitions (1 and 2) of compost treatments without and with biochar (B) are depicted individually.

Since bacterial community composition was assessed before (Werner et al., 2022), here we focus on potential human pathogens. In Figure 3 the 40 most abundant genera and species with pathogenic potential are shown. Abundances of spore-forming pathogens (see *Bacillus*; *B. cereus* and *Clostridium*; *Clostridium botulinum* in Figure 3) were very low. Strong decreases were observed for *Pseudomonas stutzeri, Stenotrophomonas maltophilia, Pseudomonas aeruginosa, Pseudomonas oryzihabitans, Pseudomonas alcaligenes, Alcaligenes faecalis, Pseudomonas syringae, Listeria monocytogenes, Staphylococcus* spp., *Enterococcus* spp., as well as for *Enterococcus faecium* and *Staphylococcus sciuri*. *Proteus mirabilis* was detected only in the start sample of one compost pile (E1B) with a relative abundance of 0.64%. The relative abundance of *Ralstonia*, *Burkholderia* and *Leishmania* species increased, as well as that of *Legionella pneumophila* and *Roseomonas gilardii*. Amplicon sequences were resolved up to the genus level and revealed the same trends for *Pseudomonas*, *Staphylococcus*, *Enterococcus*, *Proteus*, and *Legionella* spp., respectively. The other mentioned bacterial genera were not detected in the amplicon sequencing data.

### Decrease of Antibiotic Resistance Gene Abundances During Thermophilic Composting

**FIGURE 4:**
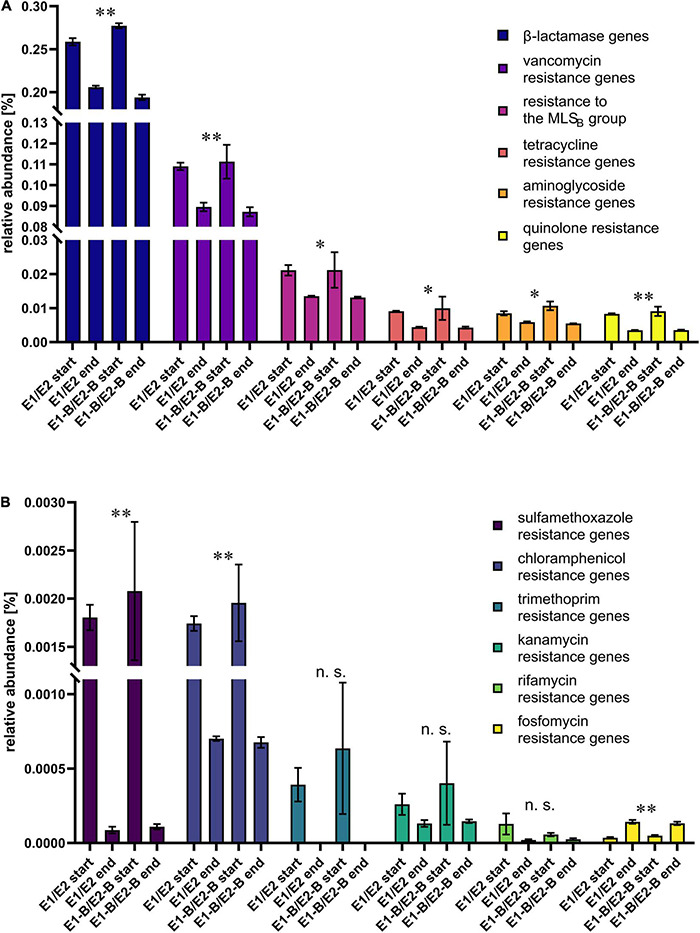
Relative abundance of resistance genes toward different antibiotic classes as assigned by KEGG database. The bars depict the mean and standard deviation of the replicate compost piles without (E1/E2) and with biochar (E1B/E2B) for start and end of composting. Higher abundant genes are depicted in **(A)**, low abundant genes in **(B)**. The numbers of assigned genes differ: 94 β-lactamase, 21 vancomycin, 19 MLS_B_, 11 tetracycline, 32 aminoglycoside, 2 quinolone, 3 sulfamethoxazole, 6 chloramphenicol, 3 trimethoprim, 2 kanamycin, 3 rifamycin and 3 fosfomycin resistance genes. Panel A shows the six highest abundant ARG groups, B the six lower abundant groups. Asterisks indicate the *p*-values obtained from Student’s *t*-test (^**^p < 0.01, *p < 0.05) representing the statistical significance of the data. Non-significant values are indicated by “n.s.” *P*-values were calculated to compare start with end samples of composting for all four compost piles combined (no differentiation of the treatments).

The presence of ARGs was assessed by means of the KEGG database. When all genes conferring resistance to the same class of antibiotic were combined, relative abundance of ARGs decreased during composting. The least abundant group of fosfomycin resistance genes was an exception, showing an increase in abundance during composting (Figure 4). Trimethoprim and sulfamethoxazole resistance genes exhibited strongest decreases of 99 and 95%, respectively, followed by the resistance genes to rifamycin with 70%, chloramphenicol with 63%, quinolones with 60%, kanamycin with 56%, tetracycline with 54%, aminoglycosides with 39%, MLS_B_ with 37%, beta-lactam antibiotics with 25% and vancomycin with 21% decreases. In addition, the relative abundance of ABC transporters and multidrug efflux pumps decreased, as well (Supplementary Figure 2).

**FIGURE 5:**
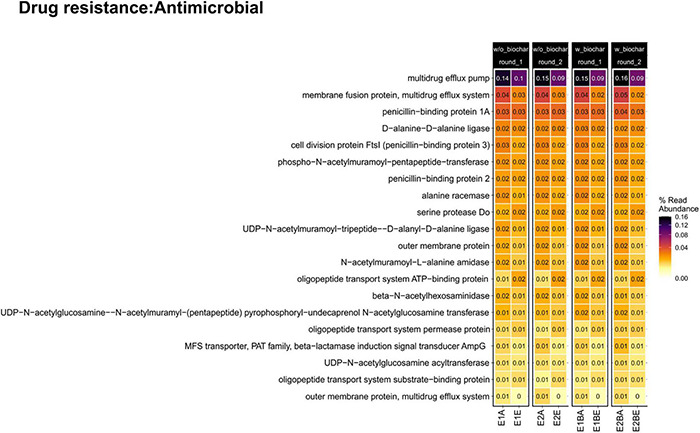
Heatmap of abundant genes conferring resistance to antibiotics in the metagenomic dataset. The compost trial comprises two repetitions (E1, E2) of two treatments without and with biochar (B) as compost supplement. Samples from start (A) and end (E) of composting are compared.

Trends of individual genes, however, differed: “Serine Protease Do” (serine endoprotease, *degP*, *htrA* [EC 3.4.21.107], involved in the two-component system in cell envelope protein folding and protein degradation, and cationic antimicrobial peptide (CAMP) resistance of periplasmic protein folding and degrading factors) and several genes involved in oligopeptide transport systems of beta-lactam resistance, ABC transporters and quorum sensing (*oppA/mppA, oppB, oppC, oppD, oppF*) showed increases from the beginning to the end of composting (Figure 5).

**FIGURE 6:**
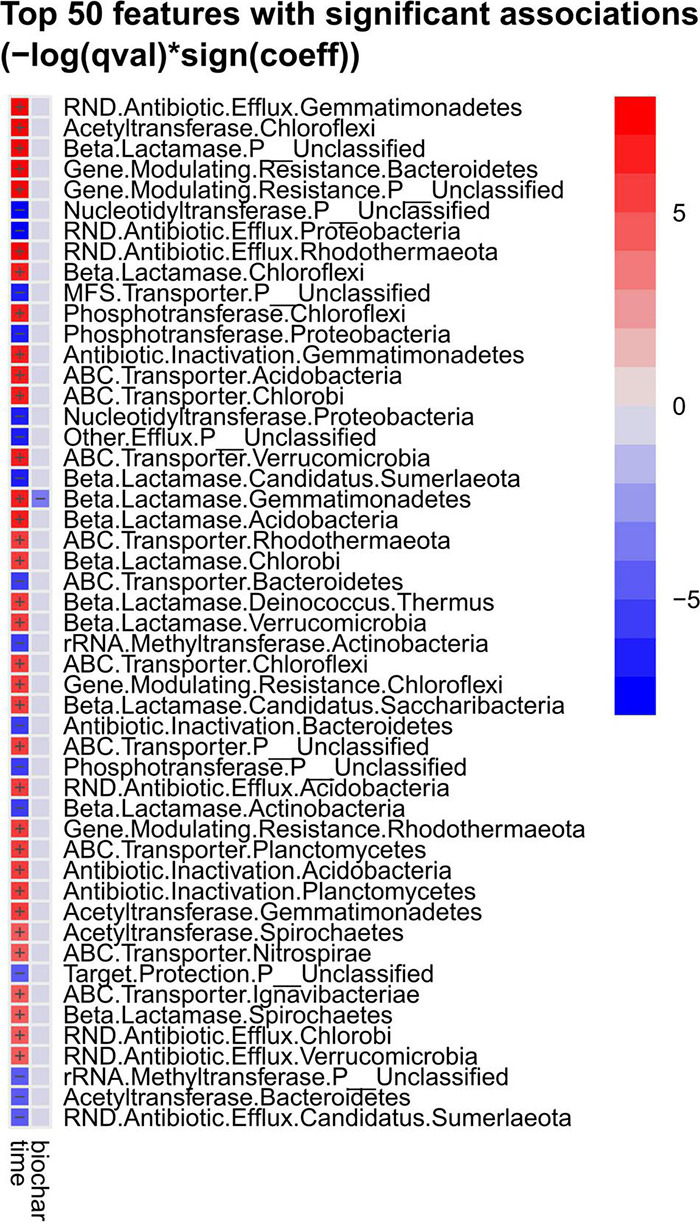
Top 50 strongly increased or decreased genes related to antibiotic resistance associated with bacterial phyla as assessed by MaAsLin2 analysis. Samples were tested regarding composting timepoint (“time” = start vs. end of composting) and regarding treatment (“biochar” = with vs. without biochar). Red color indicates increase, blue decrease of the respective gene abundance.

MaAsLin2 analysis (Mallick et al., 2021) revealed strong differences in abundance of antibiotic resistance mechanisms, assigned to a bacterial phylum, over composting and as a function of the treatment (with or without biochar) (Figure 6). Effects of the biochar treatment were observed only for beta-lactamase genes in *Gemmatimonadetes*, with lower abundance in the biochar treatment. All genes revealed differences in terms of the composting time. Beta-lactamases, ABC transporters, RND (resistance nodulation division) efflux pumps, acetyltransferases and antibiotic inactivation genes mostly increased toward the end of composting, and all entries of “gene modulating resistance” rose as well. Only phosphotransferases that modify, e.g., macrolide and aminoglycoside targets for inactivation of the antibiotic (Ramirez and Tolmasky, 2010; Golkar et al., 2018), declined in two cases. However, more resistance-related functions decreased: nucleotidyltransferases [transfer of a nucleotide monophosphate to a hydroxyl group of aminoglycoside or lincosamide antibiotics (Wright, 2005)], MFS (major facilitator superfamily) transporters (efflux pumps), rRNA methyltransferases [methylation of the antibiotic target site in the 16S ribosomal RNA (Osterman et al., 2020)] and target protection [association of a resistance protein and the antibiotic target site (Wilson et al., 2020)]. Besides unclassified phyla, ARGs associated with *Chloroflexi*, followed by *Gemmatimonadetes* and *Bacteroidetes* were the most represented taxonomic groups. *Proteobacteria* were assigned to three resistance mechanisms (RND antibiotic efflux pumps, phosphotransferases, nucleotidyltransferases), all decreased in abundance over composting. *Actinobacteria* were assigned to two resistance mechanisms that decreased (rRNA methyltransferases, beta-lactamases). Three out of four resistance functions associated with *Bacteroidetes* decreased (ABC transporters, antibiotic inactivation, acetyltransferases vs. genes modulating resistance). Apart from unclassified phyla, all other phyla were associated with increased resistance functions.

### Decrease of Abundance of Genes Involved in Type III, Type IV, and Type VI Secretion Systems

**FIGURE 7:**
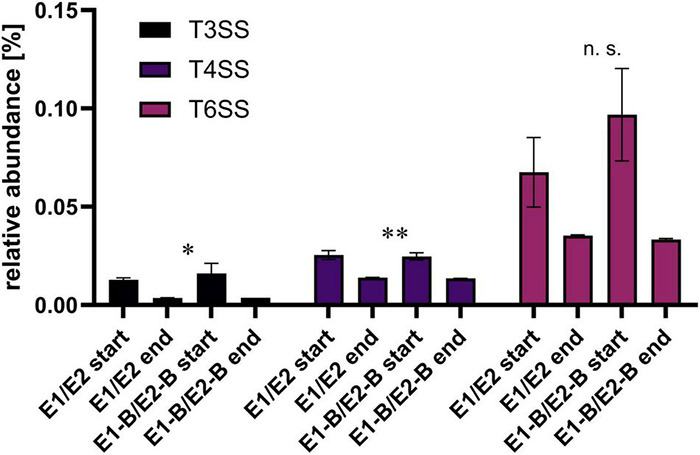
Relative abundance of type III, IV and VI secretion systems genes as assigned by KEGG database. The bars depict the mean and standard deviation of the replicate compost piles without (E1/E2) and with biochar (E1-B/E2-B) for start and end of composting. 33 genes were assigned to T3SS, 26 to T4SS and 25 toT6SS. Asterisks indicate the *p*-values obtained from Student’s *t*-test (^**^p < 0.01, *p < 0.05) representing the statistical significance of the data. Non-significant values are indicated by “n.s.” *P*-values were calculated to compare start with end samples of composting for all four compost piles combined (no differentiation of the treatments).

Concerning the potential of horizontal gene transfer and infection of eukaryotic cells, we assessed the fate of genes that are involved in type III (T3SS), IV (T4SS), and VI (T6SS) secretion systems. Relative abundance decreased for all three systems (Figure 7). Strongest decreases to around 25% of the initial level were observed for T3SS, followed by T6SS and T4SS with decreases to an average of 43 and 55%, respectively. Most individual genes of the three secretion systems followed this trend.

### Decrease in Virulence Factor Abundances During Thermophilic Composting

**FIGURE 8:**
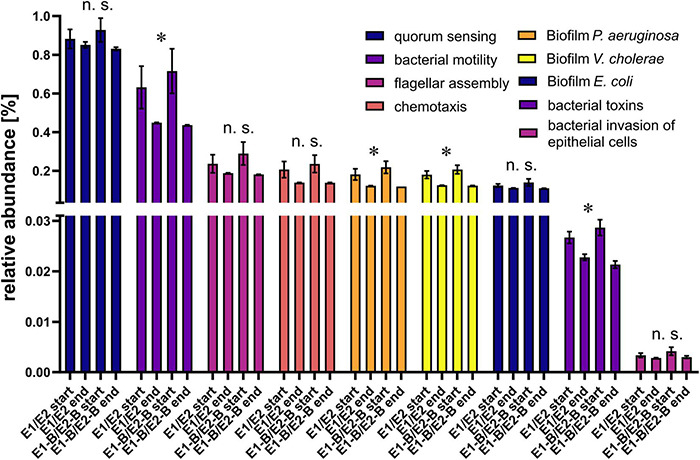
Relative abundance of genes related to quorum sensing, bacterial motility, flagellar assembly, chemotaxis, biofilm-formation for *P. aeruginosa*, *V. cholerae* and *E. coli*, bacterial toxins, and invasion of epithelial cells as assigned by KEGG database. The bars depict the mean and standard deviation of the replicate compost piles without (E1/E2) and with biochar (E1-B/E2-B) for start and end of composting. 145 genes were assigned to quorum sensing, 132 to bacterial motility, 39 to flagellar assembly, 26 to chemotaxis, 87 to biofilm-formation in *P. aeruginosa*, 78 to biofilm-formation in *V. cholerae*, 47 to biofilm-formation in *E. coli*, 40 to bacterial toxins and 65 to invasion of epithelial cells. Asterisks indicate the *p*-values obtained from Student’s *t*-test (*p < 0.05) representing the statistical significance of the data. Non-significant values are indicated by “n.s.” *P*-values were calculated to compare start with end samples of composting for all four compost piles combined (no differentiation of the treatments).

To evaluate the potential of bacterial pathogenicity before and after composting, relative abundance of genes involved in quorum sensing, bacterial motility, flagellar assembly, chemotaxis, bacterial toxins, and invasion of epithelial cells was assessed. The relative abundance of quorum sensing genes remained almost stable, whereas the other groups exhibited a decline over composting. Chemotaxis genes decreased by around 37% in abundance, bacterial motility genes showed a statistically significant decrease by 34%, flagellar assembly genes decreased by 29%, genes for invasion of epithelial cells by 22% and bacterial toxins genes exhibited significant decreases by 20% (Figure 8). Moreover, the relative abundance of biofilm-related genes for *P. aeruginosa, Vibrio cholerae* and *Escherichia coli* decreased from start to end of the composting process (Figure 8). The decrease in relative abundance of these factors for *P. aeruginosa* and *V. cholerae* was significant and more pronounced than for *E. coli*, but relative abundance levels of the samples taken at the end of composting were similar for all three species (0.11–0.13%).

### Decline of Antibiotic Resistance Gene Copy Numbers During Thermophilic Composting

**FIGURE 9:**
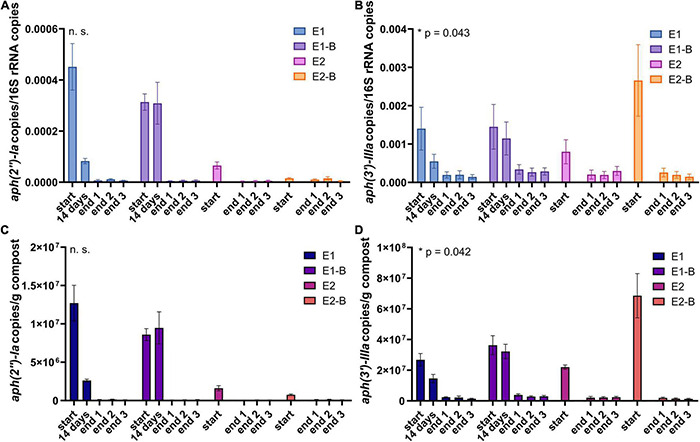
Copy numbers of aminoglycoside ARGs *aph(2″)-Ia* **(A,C)** and *aph(3**′**)-IIIa* **(B,D)** as assessed by qPCR. Copy numbers were normalized to 16S rRNA gene copy numbers (upper charts) and to 1 g compost (lower charts). Bars represent the average of three individual qPCR measurements with the standard deviation. Start, 14 days (only repetition 1) and end samples are depicted individually for the first (E1, E1-B) and second repetition of composting (E2, E2-B). End samples comprise replicates from front (end 1), middle (end 2) and back (end 3) of each compost pile. Asterisks indicate the *p*-values obtained from Student’s *t*-test (*p < 0.05) representing the statistical significance of the data. Non-significant values are indicated by “n.s.” *P*-values were calculated to compare start with end samples of composting for all four compost piles combined (average of the triplicate end samples, no differentiation of the treatments).

**FIGURE 10:**
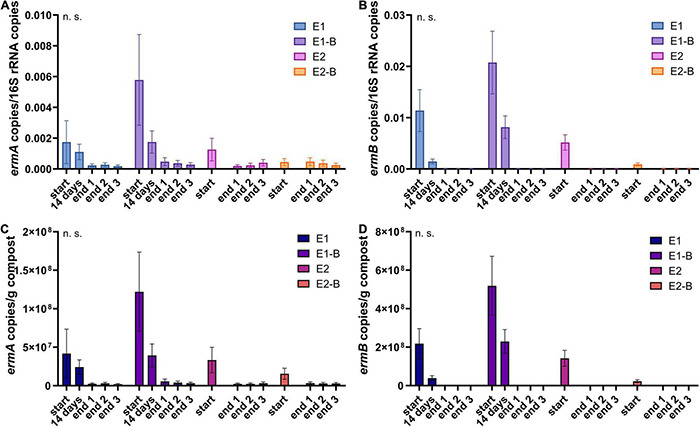
Copy numbers of erythromycin ARGs *ermA* **(A,C)** and *ermB* **(B,D)** as assessed by qPCR. Copy numbers were normalized to 16S rRNA gene copy numbers (upper charts) and to 1 g compost (lower charts). Bars represent the average of three individual qPCR measurements with the standard deviation. Start, 14 days (only repetition 1) and end samples are depicted individually for the first (E1, E1-B) and second repetition of composting (E2, E2-B). End samples comprise replicates from front (end 1), middle (end 2) and back (end 3) of each compost pile. Student’s *t*-test was conducted to test for statistical significance. Non-significant values are indicated by “n.s.” *P*-values were calculated to compare start with end samples of composting for all four compost piles combined (average of the triplicate end samples, no differentiation of the treatments).

**FIGURE 11:**
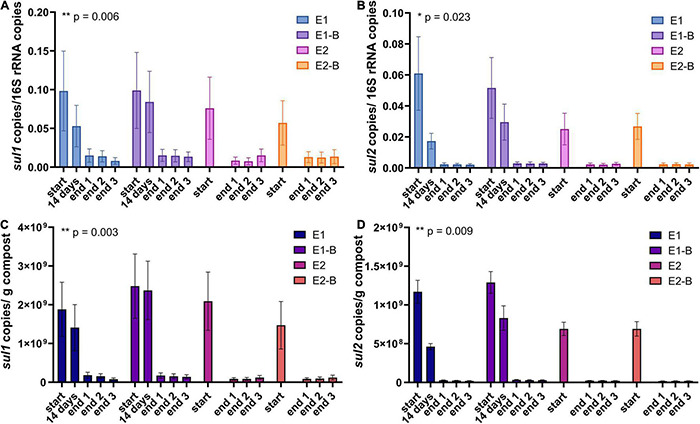
Copy numbers of sulfonamide ARGs *sul1* **(A,C)** and *sul2* **(B,D)** as assessed by qPCR. Copy numbers were normalized to 16S rRNA gene copy numbers (upper charts) and to 1 g compost (lower charts). Bars represent the average of three individual qPCR measurements with the standard deviation. Start, 14 days (only repetition 1) and end samples are depicted individually for the first (E1, E1-B) and second repetition of composting (E2, E2-B). End samples comprise replicates from front (end 1), middle (end 2) and back (end 3) of each compost pile. Asterisks indicate the *p*-values obtained from Student’s *t*-test (**p < 0.01, *p < 0.05) representing the statistical significance of the data. *P*-values were calculated to compare start with end samples of composting for all four compost piles combined (average of the triplicate end samples, no differentiation of the treatments).

**FIGURE 12:**
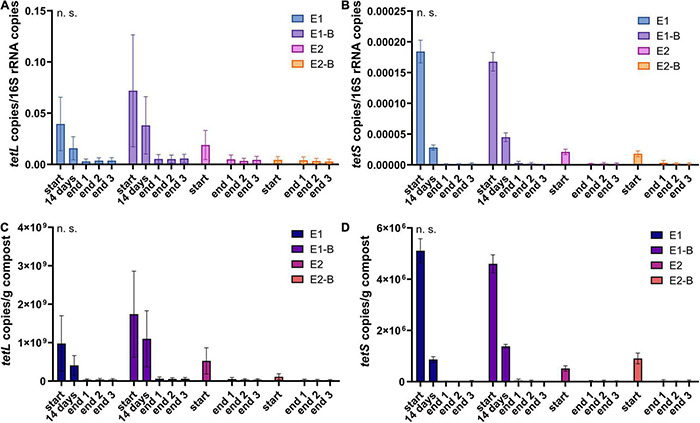
Copy numbers of tetracycline ARGs *tetL* **(A,C)** and *tetS* **(B,D)** as assessed by qPCR. Copy numbers were normalized to 16S rRNA gene copy numbers (upper charts) and to 1 g compost (lower charts). Bars represent the average of three individual qPCR measurements with the standard deviation. Start, 14 days (only repetition 1) and end samples are depicted individually for the first (E1, E1-B) and second repetition of composting (E2, E2-B). End samples comprise replicates from front (end 1), middle (end 2) and back (end 3) of each compost pile. Student’s *t*-test was conducted to test for statistical significance. Non-significant values are indicated by “n.s.” *P*-values were calculated to compare start with end samples of composting for all four compost piles combined (average of the triplicate end samples, no differentiation of the treatments).

The occurrence of individual ARGs was quantified using qPCR. Copy numbers normalized to the 16S rRNA gene and to 1 g compost showed decreases for all genes tested. The samples after 14 days of composting (available for repetition 1) exhibited decreases relative to the start samples for all ARGs tested (Figures 9–12).

The abundance of the sulfonamide resistance gene *sul1* decreased from a mean of 2.0 × 10^9^ to 1.2 × 10^8^ copies per gram compost, *sul2* from 9.6 × 10^8^ to 2.4 × 10^7^, the tetracycline resistance gene *tetL* from 8.4 × 10^8^ to 4.1 × 10^7^, *tetS* from 2.8 × 10^6^ to 2.4 × 10^4^, the aminoglycoside resistance genes *aph(3′)-IIIa* from 3.8 × 10^7^ to 2.3 × 10^6^, *aph(2″)-Ia* from 5.9 × 10^6^ to 9.4 × 10^4^, the erythromycin resistance gene *ermA* from 5.3 10^7^ to 3.1 × 10^6^, and *ermB* from 2.3 × 10^8^ to 4.1 × 10^5^ gene copies per gram compost (Supplementary Tables 5–8). Decreases of *sul1*, *sul2* and *aph(3′)-IIIa* genes were statistically significant. Strongest decreases in relative abundance from start to end of composting were observed for *ermB*, followed by *tetS*, *sul2*, *sul1*, *aph(3′)-IIIa*, *aph(2″)-Ia*, *tetL* and *ermA* (Table 1). In general, piles exhibiting peaking gene copies in the starting material were more often found in the first repetition (E1, E1-B), whereas low starting concentrations were mostly found in the second repetition (E2, E2-B).

**TABLE 1:** Decreases of gene copy numbers normalized to the 16S rRNA gene and to 1 g compost from start to end of composting.

ARG	Normalization	Decrease from start to end of composting (%)				
		E1	E1-B	E2	E2-B	Mean
*aph(2″)-Ia*	16S rRNA	98.2	98.2	93.5	25.4	**78.8**
	g compost	99.1	98.9	96.3	85.7	**95.0**
*aph(3′)-IIIa*	16S rRNA	87.2	79.5	71.0	92.5	**82.5**
	g compost	92.6	91.2	89.9	97.7	**92.8**
*ermA*	16S rRNA	87.0	93.5	78.0	18.6	**69.3**
	g compost	94.0	96.5	92.1	80.7	**90.8**
*ermB*	16S rRNA	99.6	99.8	99.2	96.6	**98.8**
	g compost	99.8	99.9	99.7	98.9	**99.6**
*sul1*	16S rRNA	87.5	85.4	86.4	77.2	**84.1**
	g compost	92.6	93.6	95.4	93.2	**93.7**
*sul2*	16S rRNA	96.4	94.4	90.6	91.3	**93.2**
	g compost	97.9	97.6	96.7	97.3	**97.4**
*tetL*	16S rRNA	91.4	92.5	77.7	23.3	**71.2**
	g compost	96.2	96.7	92.0	76.5	**90.4**
*tetS*	16S rRNA	99.3	99.0	92.2	87.5	**94.5**
	g compost	99.7	99.4	95.2	97.0	**97.8**

*Values depict the average of triplicate samples from the end of composting. A mean for all four compost piles is depicted in bold.*

## Discussion

Metagenomic sequencing was conducted on samples before and after thermophilic composting of the composting substrate (human excreta, green cuttings, and straw with and without addition of biochar) to assess possible changes in the bacterial community. We were especially interested in the fate of antibiotic resistance and bacterial pathogenicity genes.

The compost community including prokaryotes, eukaryotes, and viruses and thus its functional profile was reshaped through the composting process as suggested by PCo analyses. PCoA drew a similar picture of the dissimilarity of samples in terms of taxonomy, as well as functional genes. Differences between the treatments with and without biochar are not apparent; mature compost samples cluster close to each other indicating that the treatment had little influence on the compost community.

### Decrease of Relative Abundance of Many Human Pathogens During Thermophilic Composting

Bacteria account for most organisms in the compost. The group declined in relative abundance toward the end of composting in favor of unclassified organisms, Eukaryota and Archaea. The most abundant bacterial phyla and the changes in abundance over composting mostly correspond to the 16S rRNA gene amplicons from the same samples (Supplementary Figure 1). Differences in relative abundances of bacterial phyla might be due to possible primer and amplification biases in 16S rRNA gene amplicon sequencing (Liu et al., 2008; Větrovský and Baldrian, 2013; Yang et al., 2016; Gołębiewski and Tretyn, 2020). Besides, different databases for taxonomic classification were used that might have caused differences (Silva, NCBI).

Cultivation-based assessment of indicator organisms for human pathogens (MPN technique for *Salmonella* and *E. coli*) was described in Werner et al. (2022). *E. coli* MPNs were found to be below the German threshold values for fertilizers, whereas *Salmonella* was detected in one of the four compost piles not matching German regulations for fertilizer. Moreover, an isolation study focusing on human pathogens was performed on the mature compost. In the metagenomic study, the relative abundance of human pathogenic microorganisms mostly declined during composting. Several *Pseudomonas* species showed a strong decrease in relative abundance, as well as *A. faecalis*, *S. maltophilia*, *L. monocytogenes*, *E. faecium*, *Enterococcus* spp., and *Staphylococcus* spp. *Pseudomonas* species and *S. maltophilia* are known pathogens. They are mostly opportunistic and have been isolated from diverse habitats, like soil, the rhizosphere and water (Aujoulat et al., 2012; Brooke, 2012; De-la-Peña and Loyola-Vargas, 2014). *A. faecalis* is a rather uncommon nosocomial pathogen that has also been isolated from soil, water, or the human gut. It has also been found in compost, where it is involved in nitrification and denitrification processes (Tena et al., 2015; Chen et al., 2021). *L. monocytogenes*, a common foodborne pathogen, present in soil and aquatic environments, is especially harmful to elderly or immunocompromised patients (Schlech, 2019). Enterococci and staphylococci pose a high risk to public health due to strains harboring resistances to many different antibiotic classes. Vancomycin-resistant *E. faecium* and methicillin-resistant and vancomycin- intermediate and -resistant *S. aureus* were listed as high priority pathogens for research and development of new antibiotics by the WHO (Tacconelli et al., 2018).

Species increasing in relative abundance during composting belong to the genera *Ralstonia*, *Burkholderia*, and *Leishmania*. The first are rather known as environmental bacteria, although few species can cause disease in patients with preconditions that are often difficult to treat due to multiple resistances (Ryan and Adley, 2014). *Burkholderia* is likewise considered as an environmental clade of mostly saprophytic species with high potential for bioremediation due to the degradation of aromatic compounds. *B. mallei* is the only obligate pathogen, several others are known as opportunistic and are often resistant to several antimicrobials (Rhodes and Schweizer, 2016; Morya et al., 2020). *Ralstonia* and especially *Burkholderia* spp. have been found dominant during poultry manure composting as assessed by 16S rRNA gene DGGE (Omar et al., 2015). *Leishmania* is a genus of obligate protozoan parasites with a lifecycle in sand flies and mammals. Approximately 20 species are pathogenic to humans. The increase in *Leishmania* DNA during composting is interesting, since the organism needs a host to proliferate. One explanation could be infected rodents that cause entry of *Leishmania* or their genes into the compost heaps (Akhoundi et al., 2016). Another reason could be breeding sandflies. Breeding and larvae habitats of sandflies are not well understood. Eggs have been found in diverse non-aquatic environments, usually moist habitats rich in organic matter (Killick-Kendrick, 1999; Feliciangeli, 2004; Vivero et al., 2015).

*L. pneumophila* is the etiological agent of the Legionnaires’ disease and Pontiac fever and is usually found in water systems and aquatic environments. Biofilms or a parasitic lifestyle in amoeba, protozoa or nematode hosts allow the species to survive in natural habitats. Moreover, *L. pneumophila* can change between a transmissive and a replicative phase. The latter takes place in nutrient-rich settings, which might explain the rise of abundance of this microorganism in our study (Abu Khweek and Amer, 2018). Besides, temperatures of 45–50°C were found optimal for *L. pneumophila* in water distribution systems and only temperatures of 55°C prevent outbreaks (Lin et al., 1998; Darelid et al., 2002). *Legionella* spp., amongst others *L. pneumophila*, have been isolated from different green waste composts particularly in low-temperature composts (Casati et al., 2010; Conza et al., 2013). It can therefore be assumed that the poor temperature development in the compost trial with peak temperatures between 51 and 57°C was insufficient to reduce the abundance of *L. pneumophila*. *Roseomonas gilardii* is a rare opportunistic pathogen causing bacteremia with low morbidity and mortality in humans. It was originally isolated from drinking water and has since then mostly been described in clinical samples. The natural habitat is not known, the bacterium might, however, be associated with humans as a commensal. Due to its ability to utilize urea, the compost environment could have favored its growth (Rihs et al., 1993; Shokar et al., 2002; Schlappi et al., 2020).

The metagenomic data suggested the putative presence of several human pathogens. Severe pathogens showed a decline during composting. Mostly environmental genera with few opportunistic representatives or species less common as pathogens (besides *L. pneumophila*) increased in relative abundance over composting. *L. pneumophila* growth was presumably linked to poor temperature development during the composting trial. The treatments with and without biochar did not exhibit differences regarding the microbial community or pathogenic strains.

### Thermophilic Composting as an Effective Treatment to Decrease Bacterial Pathogenicity and the Dissemination of Antibiotic Resistance Genes

ARGs of major classes of antibiotics decreased in relative abundance from start to end of composting. In a recently published, comprehensive study Keenum et al. (2021) compared start and mature compost from dairy and cattle manure under antibiotic administration and without administration. They analyzed relative abundance (16S rRNA gene normalized) of ARGs using metagenomic sequencing. Strongest decreases from start to end of composting were observed for trimethoprim, tetracycline and MLS_B_ resistance genes. In our study, trimethoprim resistance genes also exhibited strongest decreases of 99% from start to end, followed by sulfamethoxazole and rifamycin resistance genes. Tetracycline (54%) and MLS_B_ resistance genes (37%) showed less pronounced declines in relative abundance through composting.

In a metagenomic study on agricultural soils fertilized with untreated and composted cattle manure, total relative ARG abundance did not reveal differences. However, application of composted manure reduced the ARG diversity in the soil by 21%, whereas increases in diversity were detected for raw manure application (Wind et al., 2021). Guron et al. (2019) compared ARG abundance and diversity on lettuce and radish fertilized with raw and composted cattle manure, and fertilizer as a control. Vegetable type had the strongest influence on the ARG abundance and diversity, followed by soil type. Composting as a pre-treatment resulted in a different ARG profile, but not in different relative abundances when compared with raw manure. Another metagenomic study of ARGs on vegetables grown in soil treated with raw or composted chicken litter revealed decreased relative abundance of total ARGs for compost-application, despite increases of specific ARGs (Subirats et al., 2021). Although fertilization with compost differs from the composting process itself and therefore cannot be compared directly, the field trials with raw vs. composted manure show interesting similarities with our study (starting material vs. finished compost). In our study, the reduction of ARGs differed for the different classes of ARGs, which likewise changed the resistance profile from start to end of the composting process, during which the total relative ARG abundance decreased. Single genes exhibited a contrary tendency, which agrees with the findings on compost-fertilized vegetables by Subirats et al. (2021). Metagenomic analyses on animal manure compost and its influence on agricultural soils and vegetables show advantages of composting over raw manure application in terms of ARG abundance and diversity.

qPCR results on the fate of individual ARGs during animal manure composting differ in the literature. Decreases were reported for beta-lactam, macrolide, sulfonamide, tetracycline and quinolone resistance genes during swine and poultry manure composting (Wang et al., 2016; Guo et al., 2017; Chen Z. et al., 2018; Esperón et al., 2020), while increases in aminoglycoside, tetracycline and sulfonamide resistance genes (especially *sul1*) were detected during cattle, swine and chicken manure composting (Li et al., 2017; Cheng et al., 2019; Duan et al., 2019; Esperón et al., 2020). Studies on sewage sludge composting detected a variety of beta-lactam, macrolide, sulfonamide, tetracycline and quinolone resistance genes with different tendencies of increase or decrease during the composting process (Jang et al., 2018; Liao et al., 2018; Cui et al., 2020; Wei et al., 2020). Mechanisms of ARG reduction in animal raw and treated waste are mostly unknown and should be focused on in future research (Pereira et al., 2021).

The marker genes analyzed in the present study showed strong decreases of copy numbers during composting (> 90% for all genes normalized to 1 g compost, > 70% when normalized to 16S rRNA gene copy numbers). Copy numbers of the same gene usually ranged within the same order of magnitude in the end samples despite different levels in the start samples. Highest decreases of around 99% were observed for erythromycin resistance gene *ermB*, which also exhibited low copy numbers in the end samples (10^4^–10^5^/g compost). This is in accordance with conventional PCR results from the same samples, which showed *ermB* in the start samples and after 14 days of composting but could not detect the gene in the end samples (Werner et al., 2022). *ermB* is a transferable erythromycin resistance gene present in many clinically relevant *Enterobacteriaceae*, in which it is often found on plasmids carrying different *erm* genes (Roberts, 2004; Gomes et al., 2017). *ermA* on the other hand was detected in all samples using conventional PCR (Werner et al., 2022), and still exhibited copy numbers of 10^6^ in the end samples. The gene is associated with transposons, and frequently found in *S. aureus*, including MRSA, where it is often integrated in its chromosome (Kadlec et al., 2012; Sarrou et al., 2019). Besides Staphylococci, *Actinobacillus*, *Peptostreptococcus*, and *Streptococcus* host the *ermA* gene (Roberts, 2004). All these genera are represented in the metagenomic data in both start and end samples (data not shown), which might explain the comparatively high copy numbers in the mature compost.

The relative abundance of the tetracycline resistance gene *tetL* decreased least over composting and showed rather high copy numbers of 10^7^/g compost in the end samples, compared with other genes analyzed. Correspondingly, *tetL* detection *via* the less sensitive conventional PCR revealed positive results for both start and end samples with few exceptions (Werner et al., 2022). The TetL protein functions as an antiporter not only for tetracycline, but also for sodium and potassium ions and is therefore beneficial for K^+^ acquisition and during Na^+^ and alkali stresses (Krulwich et al., 2001). This multifunctional role might explain the high level of *tetL* after composting. In addition, the gene is not only present on plasmids, but was also detected in the genome of *Bacillus subtilis* (Krulwich et al., 2001). 16S rRNA gene amplicon sequencing of the same sample set revealed *Bacilli* as the class highest in relative abundance, and *B. subtilis* as the most frequently isolated species from mature compost (Werner et al., 2022). TetS follows a different mode of action as the antiporter TetL. It functions as a protection protein at the ribosomal binding site of tetracycline antibiotics. It was found in both Gram-positive and Gram-negative genera, all of which have been detected in the metagenome (data not shown): *Citrobacter*, *Enterococcus*, *Klebsiella*, *Lactobacillus*, *Lactococcus*, *Listeria*, *Staphylococcus*, *Streptococcus*, and *Veillonella.* However, the gene was not originally associated with mobile genetic elements and is not among the frequently detected *tet*-genes (Roberts, 2005; Roberts and Schwarz, 2016). This corresponds with lowest abundances among all analyzed genes in both the start material and the mature compost. The same trends were found using conventional PCR: *tetS* was only detected in E1 and E1-B in the start and 14-days samples (Werner et al., 2022).

qPCR revealed decreases of *aph(2″)-Ia* to comparatively low abundances of 10^4^ copies/g compost in the mature compost. The gene is located downstream of *aac(6′)-Ie* on Tn*5281* which is often localized on conjugative plasmids resulting in a bifunctional enzyme *aac(6′)-Ie-aph(2″)-Ia* conferring resistance to most aminoglycoside antibiotics (Vakulenko and Mobashery, 2003; Hegstad et al., 2010; Rosvoll et al., 2012). It is associated with *Enterococcus*, *Streptococcus*, and *Staphylococcus* spp. (Vakulenko and Mobashery, 2003), which were found to decrease in the metagenome. Kanamycin/amikacin resistance gene *aph(3′)-IIIa* revealed comparatively low levels in both start and end samples with intermediate decreases, which matches the PCR results showing *aph(3′)-IIIa* in samples taken at the start and after 14 days of composting, but not in samples taken at the end (Werner et al., 2022). Aph(3′) is the largest group of clinically relevant aminoglycoside phosphotransferases. *aph(3′)-III* has been frequently found in pathogens like enterococci and staphylococci, including MRSA, and is often located in the vicinity of genes conferring resistance to other antibiotics (Vakulenko and Mobashery, 2003).

Conventional PCR analysis of the sulfonamide resistance genes *sul1* and *sul2* was positive for all samples (Werner et al., 2022). Using qPCR, both genes exhibited significant decreases during composting. Before composting, gene copy numbers were high for both genes (10^8^–10^9^/g compost). *sul1* was still present with an average of 10^8^ copies/g compost in the mature compost, whereas *sul2* was reduced to 10^7^ copies/g compost. *sul1* and *sul2* are located on transposons and plasmids of Gram-negative bacteria and are often associated with other resistances. Their dissemination is therefore very effective, which might be a reason that the genes have often been found after composting, as well (Enne et al., 2001; Sköld, 2001). Besides, the sulfonamide sulfamethazine is harder to degrade through composting than other antibiotics. Thus, selective pressure maintains and promotes the persistence of *sul* genes (Youngquist et al., 2016).

The qPCR results complement our conventional PCR study (Werner et al., 2022) by demonstrating pronounced decreases of clinically relevant resistance genes for gentamicin, kanamycin/amikacin, sulfonamide, and tetracycline during thermophilic composting of human excreta. Differences between the two treatments with and without biochar were not observed, possibly due to rather low biochar contents. Studies comparing different biochar contents found stronger decreases of ARG abundances in composts containing biochar than in composts without biochar. However, increments of the biochar content did not significantly improve ARG decreases. This was shown for swine manure compost with 6, 12, and 24% biochar, where 6% biochar improved ARG removal and higher percentages revealed the opposite effect (Wang et al., 2018). Li et al. (2017) compared chicken manure compost supplemented with 0, 5, 10, and 20% bamboo charcoal. 10% biochar was found to achieve the best results regarding ARG removal (Li et al., 2017). In a recent study on municipal sludge three treatments apart from the control without additives were tested: addition of hyperthermophiles, hyperthermophiles with 2% biochar, hyperthermophiles with 5% biochar. Biochar significantly increased ARG removal rates, but no significant differences between the different biochar contents were found (Fu et al., 2021). Further research would be needed to understand the mechanisms behind different biochar or compost types and biochar contents on ARG removal.

T4SSs enable horizontal gene transfer through conjugation in Gram-negative and Gram-positive bacteria, as well as in some archaea. They have been encountered in most bacterial species and are therefore crucial, when studying horizontal gene transfer in clinically relevant bacteria like *Enterococcus*, *Clostridium, Staphylococcus*, or *Streptococcus* spp. Beside their role in the dissemination of ARGs, T4SSs are involved in the transfer of virulence factors, in biofilm formation, competition with other microbes through killing toxins, and injection of effector proteins into host cells (Grohmann et al., 2003, 2018; Goessweiner-Mohr et al., 2014). The decrease in relative abundance of T4SS genes over composting in the present study indicates that the potential for horizontal gene transfer, as well as bacterial pathogenicity is reduced. This has been shown for manure composts before by quantifying the integrase gene *intI1* and transposase genes as markers for horizontal gene transfer (Wang et al., 2021; Zhou et al., 2021). Biochar addition to the compost increased *intI1* removal efficiency during composting (Zhou et al., 2021). This might be due to higher temperatures and a longer thermophilic phase through biochar (Godlewska et al., 2017). The importance of high temperatures for removal efficiencies of integrase genes during composting was demonstrated by Qian et al. (2016). In the present study biochar did not increase temperatures during the thermophilic phase, and temperature development was poor in general, which might explain that relative T4SS gene abundances did not differ between the treatments with and without the addition of biochar.

The decrease in pathogenicity was further demonstrated by the decrease in genes associated with T3SSs and T6SSs in our study. T3SSs are common in many Gram-negative bacteria and have often been acquired through horizontal gene transfer. Pathogens like *Pseudomonas*, *Yersinia*, *Shigella* or *E. coli* use T3SSs to inject effector proteins into eukaryotic cells (Büttner, 2012; Green and Mecsas, 2016). Functional diversity of T6SSs could explain their comparatively high relative abundance in start and end samples, respectively. The primary function of T6SSs is effector protein translocation to competing bacteria of the same or different species, and fungi. They are also involved in transformation of naturally competent bacteria and aid in metal scavenging providing survival benefits. In addition, effector proteins can be translocated from bacteria to eukaryotic cells and thereby they contribute to bacterial virulence (Borgeaud et al., 2015; Green and Mecsas, 2016; Coulthurst, 2019).

Virulence factors comprise a broad group of functions that are used by pathogens to cause infection and enhance their pathogenicity. They include toxin production, motility, adhesins, capsules, quorum sensing and biofilm formation, as well as secretion systems. Strategies are diverse, complex and virulence factor categories are not clearly defined, e.g., quorum sensing being essential for bacteria to form biofilms (Casadevall and Pirofski, 2009; Hill, 2012).

We assessed the abundance of the functional groups, quorum sensing, bacterial motility, flagellar assembly, chemotaxis, toxins, invasion of epithelial cells, and biofilm formation, and found decreases in relative abundance for all groups of genes. Studies on compost and virulence mainly focus on plant pathogenicity rather than on the impact on human health. They usually assess plant-waste compost, which is not well comparable to our study (Lim et al., 2014; Antoniou et al., 2017; Andreazza et al., 2019). Indicator organisms for plant pathogenicity according to German and European regulations for fertilizer comprise the genus *Tobamovirus*, especially Tobacco mosaic viruses, the clubroot pathogen *Plasmodiophora brassicae*, as well as the fungal species *Synchytrium endobioticum*, *Rhizoctonia solani*, and *Sclerotinia* spp. (BioAbfV, 1998; Carrington, 2001; DüMV, 2012). All these indicators were detected in the start and mature compost samples (data not shown).

Sidhu et al. (2017) compared raw and treated sludge from a wastewater treatment plant and found decreases of microbial genes involved in signal transduction, cell motility and chemotaxis. Although the treatment was different from our study, the results indicate that fecal material is usually rich in potential virulence factors that decline through treatment changing the conditions for the microbial community. As virulence factors are not solely used by pathogenic microorganisms, it is not likely that the respective genes disappear completely (Hill, 2012). In fact, bacterial secretion systems or some classes of ARGs exhibited stronger decreases than other groups of virulence genes. Especially the relative abundance of quorum sensing genes exhibited only minor declines. This is most likely due to its involvement in numerous bacterial processes that are not exclusively used for infection, but also for competition and cooperation with other microbes (Zhao et al., 2020). *N*-Acyl homoserine lactone (AHL)-mediated quorum sensing has also been described in soils supplemented with compost, which might be a hint for the natural occurrence of quorum sensing systems in compost (Burmølle et al., 2005).

In summary, we have conducted a metagenomic analysis of compost samples before and after thermophilic composting of human excreta from dry toilets together with green cuttings and straw, with and without biochar, to assess the influence of the process on human pathogens, antibiotic resistance, and bacterial virulence. In terms of pathogenicity and antibiotic resistance, the addition of biochar had no positive effect. Severe pathogenic species decreased in relative abundance, the reverse tendency was generally found in less pathogenic or opportunistic species. The overall level of ARGs was lower after composting. Resistance gene analyses using qPCR showed strong decreases of genes conferring resistance to erythromycin, aminoglycosides, sulfonamide, and tetracyclines over composting. Declines in the relative abundance of T4SSs indicate that the potential for horizontal gene transfer was also reduced. Pathogenicity strategies like T3SSs, biofilm formation capacity, chemotaxis or bacterial motility also exhibited decreases in relative abundance, thereby suggesting that the infection potential of the bacterial community was reduced through composting. All these strategies are also used by non-pathogenic bacteria to compete with the surrounding microbes. Thus, it would be interesting to assess a natural “background” level in a non-anthropogenic environment by evaluating the impact of compost fertilization in comparison to other organic fertilizers concerning the potential spread of antibiotic resistances. Since the study used DNA-based analysis, the results reflect the general potential of the compost community, but not the actually expressed functions. Metatranscriptomic analysis would be interesting to assess how the composting process changes functional gene expression.

## Data Availability Statement

The datasets presented in this study can be found in online repositories. The names of the repository/repositories and accession number(s) can be found in Supplementary Table S1: NCBI—PRJNA782422.

## Author Contributions

EG and KW designed and supervised the laboratory experiments. KW, DS, AP, ND, LF, and KA performed the laboratory experiments and analyzed the data. KP, NB, and TH designed the composting trial. TH conducted process control. KW, DS, AP, TH, and EG wrote the manuscript and designed all figures and tables. RD contributed with insightful discussions on analysis and interpretation of the data. All authors interpreted the results, read, revised the manuscript, contributed to the article, and approved the submitted version.

## Conflict of Interest

The authors declare that the research was conducted in the absence of any commercial or financial relationships that could be construed as a potential conflict of interest.

## Publisher’s Note

All claims expressed in this article are solely those of the authors and do not necessarily represent those of their affiliated organizations, or those of the publisher, the editors and the reviewers. Any product that may be evaluated in this article, or claim that may be made by its manufacturer, is not guaranteed or endorsed by the publisher.

## Footnotes

1 hmmsearch, http://hmmer.org/
