# Supplementary material for: Metagenomic Insights Into the Changes of Antibiotic Resistance and Pathogenicity Factor Pools Upon Thermophilic Composting of Human Excreta
Source: Front Microbiol. 2022 Mar 31;13:826071. doi: 10.3389/fmicb.2022.826071 (PMC9009411; doi:10.3389/fmicb.2022.826071)
Supplement: Supplementary file 1 [file Data_Sheet_1.docx]

Table S1. Accession numbers for the metagenomic sequences (library strategy: WGS, library source: metagenomic, library selection: random, library layout: paired, platform: Illumina, instrument model: Illumina HiSeq 2500).

| accession number | study number | bioproject accession number | biosample accession number | library ID | title | design description | file-type | filename | filename2 | filename3 | filename4 |
| --- | --- | --- | --- | --- | --- | --- | --- | --- | --- | --- | --- |
| SRR17041395 | SRP347939 | PRJNA782422 | SAMN23382798 | E1A_mg | Shotgun sequencing of the metagenomic DNA of sample E1A | Metagenomic DNA was extracted from compost of dry toilets. Prepared libraries (Nextera Flex) were sequenced on an Illumina HS2500 system | fastq | 20200302_AGEG_m_1_S5_L001_R1_001.fastq.gz | 20200302_AGEG_m_1_S5_L001_R2_001.fastq.gz | 20200302_AGEG_m_1_S5_L002_R1_001.fastq.gz | 20200302_AGEG_m_1_S5_L002_R2_001.fastq.gz |
| SRR17041394 | SRP347939 | PRJNA782422 | SAMN23382793 | E1BA_mg | Shotgun sequencing of the metagenomic DNA of sample E1BA | Metagenomic DNA was extracted from compost of dry toilets. Prepared libraries (Nextera Flex) were sequenced on an Illumina HS2500 system | fastq | 20200302_AGEG_m_2_S6_L001_R1_001.fastq.gz | 20200302_AGEG_m_2_S6_L001_R2_001.fastq.gz | 20200302_AGEG_m_2_S6_L002_R1_001.fastq.gz | 20200302_AGEG_m_2_S6_L002_R2_001.fastq.gz |
| SRR17041392 | SRP347939 | PRJNA782422 | SAMN23382804 | E2A_mg | Shotgun sequencing of the metagenomic DNA of sample E2A | Metagenomic DNA was extracted from compost of dry toilets. Prepared libraries (Nextera Flex) were sequenced on an Illumina HS2500 system | fastq | 20200302_AGEG_m_3_S7_L001_R1_001.fastq.gz | 20200302_AGEG_m_3_S7_L001_R2_001.fastq.gz | 20200302_AGEG_m_3_S7_L002_R1_001.fastq.gz | 20200302_AGEG_m_3_S7_L002_R2_001.fastq.gz |
| SRR17041391 | SRP347939 | PRJNA782422 | SAMN23382800 | E2BA_mg | Shotgun sequencing of the metagenomic DNA of sample E2BA | Metagenomic DNA was extracted from compost of dry toilets. Prepared libraries (Nextera Flex) were sequenced on an Illumina HS2500 system | fastq | 20200302_AGEG_m_4_S8_L001_R1_001.fastq.gz | 20200302_AGEG_m_4_S8_L001_R2_001.fastq.gz | 20200302_AGEG_m_4_S8_L002_R1_001.fastq.gz | 20200302_AGEG_m_4_S8_L002_R2_001.fastq.gz |
| SRR17041390 | SRP347939 | PRJNA782422 | SAMN23431679 | E1E_mg | Shotgun sequencing of the metagenomic DNA of sample E1E | Metagenomic DNA was extracted from compost of dry toilets. Prepared libraries (Nextera Flex) were sequenced on an Illumina HS2500 system | fastq | 20200302_AGEG_m_5_S9_L001_R1_001.fastq.gz | 20200302_AGEG_m_5_S9_L001_R2_001.fastq.gz | 20200302_AGEG_m_5_S9_L002_R1_001.fastq.gz | 20200302_AGEG_m_5_S9_L002_R2_001.fastq.gz |
| SRR17041389 | SRP347939 | PRJNA782422 | SAMN23431680 | E1BE_mg | Shotgun sequencing of the metagenomic DNA of sample E1BE | Metagenomic DNA was extracted from compost of dry toilets. Prepared libraries (Nextera Flex) were sequenced on an Illumina HS2500 system | fastq | 20200302_AGEG_m_6_S10_L001_R1_001.fastq.gz | 20200302_AGEG_m_6_S10_L001_R2_001.fastq.gz | 20200302_AGEG_m_6_S10_L002_R1_001.fastq.gz | 20200302_AGEG_m_6_S10_L002_R2_001.fastq.gz |
| SRR17041388 | SRP347939 | PRJNA782422 | SAMN23431681 | E2E_mg | Shotgun sequencing of the metagenomic DNA of sample E2E | Metagenomic DNA was extracted from compost of dry toilets. Prepared libraries (Nextera Flex) were sequenced on an Illumina HS2500 system | fastq | 20200302_AGEG_m_7_S11_L001_R1_001.fastq.gz | 20200302_AGEG_m_7_S11_L001_R2_001.fastq.gz | 20200302_AGEG_m_7_S11_L002_R1_001.fastq.gz | 20200302_AGEG_m_7_S11_L002_R2_001.fastq.gz |
| SRR17041387 | SRP347939 | PRJNA782422 | SAMN23431682 | E2BE_mg | Shotgun sequencing of the metagenomic DNA of sample E2BE | Metagenomic DNA was extracted from compost of dry toilets. Prepared libraries (Nextera Flex) were sequenced on an Illumina HS2500 system | fastq | 20200302_AGEG_m_8_S12_L001_R1_001.fastq.gz | 20200302_AGEG_m_8_S12_L001_R2_001.fastq.gz | 20200302_AGEG_m_8_S12_L002_R1_001.fastq.gz | 20200302_AGEG_m_8_S12_L002_R2_001.fastq.gz |

Table S2. Forward and reverse read length, read count, number of base pairs and GC content of metagenomic reads before and after quality filtering (QF). Samples comprised samples from start and end of two composting repetitions (E1/E2) of two treatments each (without and with biochar (B)).

| Sample | Forward read length (before QF) | Reverse read length (before QF) | Read count (before QF) | Base pairs (before QF) | GC content (before QF) | Forward read length  (after QF) | Reverse read length  (after QF) | Read count (after QF) | Base pairs (after QF) | GC content (after QF) |
| --- | --- | --- | --- | --- | --- | --- | --- | --- | --- | --- |
| E1 start | 238 | 238 | 44,872,702 | 1.0722E+10 | 0.605821 | 239 | 238 | 44090688 | 1.053E+10 | 0.606537 |
| E1-B start | 236 | 236 | 52,093,984 | 1.233E+10 | 0.592856 | 236 | 236 | 50950114 | 1.2056E+10 | 0.593721 |
| E2 start | 237 | 237 | 57,943,718 | 1.3771E+10 | 0.613473 | 237 | 237 | 56984026 | 1.3542E+10 | 0.614155 |
| E2-B start | 236 | 236 | 49,980,712 | 1.1803E+10 | 0.590313 | 236 | 236 | 49042284 | 1.1588E+10 | 0.591072 |
| E1 end | 239 | 239 | 57,012,384 | 1.3636E+10 | 0.635177 | 239 | 238 | 55974358 | 1.3376E+10 | 0.636092 |
| E1-B end | 237 | 237 | 79,650,442 | 1.8908E+10 | 0.632174 | 237 | 237 | 78211956 | 1.8563E+10 | 0.633127 |
| E2 end | 239 | 239 | 94,600,400 | 2.2676E+10 | 0.639093 | 239 | 239 | 93059152 | 2.2287E+10 | 0.639957 |
| E2-B end | 238 | 238 | 51,576,544 | 1.2294E+10 | 0.640451 | 238 | 238 | 50728850 | 1.2084E+10 | 0.6414 |

Table S3. qPCR primers and probes with target gene, sequence, amplicon size, positive control for generation of standards, and primer/probe reference. All programs were initiated with a denaturation step of 95 °C for 10 min. Fluorescence was measured during the 72 °C/1s step of the qPCR program. Standard curves of 10^1^ to 10^8^ gene copies were used for quantification.

| Target gene | Primer/Probe | Sequence (5’🡪3’) | Amplicon size [bp] | Positive Control | Reference | qPCR program  (45 cycles) | |
| --- | --- | --- | --- | --- | --- | --- | --- |
| 16S rRNA | Bact348f | AGGCAGCAGTDRGGAAT | 439 | *Escherichia coli* K12 C600 | Takai & Horikoshi, (2000) | 95 °C | 10 s |
|  | Bact786r | GGACTACYVGGGTATCTAAT |  |  |  | 50 °C | 40 s |
|  | tpBact | 6FAM-TGCCAGCAGCCGCGGTAATACRDAG-TAMRA |  |  |  | 72 °C | 1 s |
| *aph(2’’)-Ia* | q_aph(2‘‘)-Ia-F | GCAGTTATTTTAGACCCTCA | 174 | *Staphylococcus aureus* SK5428 | this study | 95 °C | 10 s |
|  | q_aph(2‘‘)-Ia-R | CATTGCCTTAACATTTGTGG |  |  |  | 57 °C | 30 s |
|  | q_aph(2‘‘)-Ia-P | 6FAM- GCCAGAACATGAATTACACGAGGGCA -TAMRA |  |  |  | 72 °C | 1 s |
| *aph(3’)-IIIa* | q_aph(3‘)-IIIa-F | ACATATCGGATTGTCCCTATACGAA | 82 | *Enterococcus faecalis* RE25 | Woegerbauer et al. (2014) | 95 °C | 10 s |
|  | q_aph(3‘)-IIIa-R | TCGGCCAGATCGTTATTCAGTA |  |  |  | 60 °C | 20 s |
|  | q_aph(3‘)-IIIa-P | 6FAM-AGACAGCCGCTTAGCCGAATTGGATT-TAMRA |  |  |  | 72 °C | 1 s |
| *ermA* | q_ermA-F | TCTTATCGTTGAGAAGGGAT | 137 | *Staphylococcus aureus* MRSA 04-02981 | this study | 95 °C | 10 s |
|  | q_ermA-R | CTACACTTGGCTTAGGATGA |  |  |  | 55 °C | 30 s |
|  | q_ermA-P | 6FAM-TGCAAAATCTGCAACGAGCTTTGGG-TAMRA |  |  |  | 72 °C | 1 s |
| *ermB* | q_ermB-F | GGATTCTACAAGCGTACCTTGGA | 92 | *Enterococcus gallinarum* SF9117 | Böckelmann et al. (2009) | 95 °C | 15 s |
|  | q_ermB-R | GCTGGCAGCTTAAGCAATTGCT |  |  |  | 60 °C | 60 s |
|  | q_ermB-P | 6FAM–CACTAGGGTTGCTCTTGCACACTCAAGTC–TAMRA |  |  |  | 72 °C | 1 s |
| *sul1* | q_sul1-F | CCGTTGGCCTTCCTGTAAAG | 66 | *Escherichia coli* K12 J53 | Heuer & Smalla (2007) | 95 °C | 10 s |
|  | q_sul1-R | TTGCCGATCGCGTGAAGT |  |  |  | 58 °C | 30 s |
|  | q_sul1-P | 6FAM-CAGCGAGCCTTGCGGCGG-TAMRA |  |  |  | 72 °C | 1 s |
| *sul2* | q_sul2-F | CGGCTGCGCTTCGATT | 59 | *Escherichia coli* PS84 | Heuer et al. (2008) | 95 °C | 15 s |
|  | q_sul2-F | CGCGCGCAGAAAGGATT |  |  |  | 53 °C | 10 s |
|  | q_sul2-P | 6FAM-CGGTGCTTCTGTCTGTTTCGCGC-TAMRA |  |  |  | 60 °C | 60 s |
|  |  |  |  |  |  | 72 °C | 1 s |
| *tetL* | q_tetL-F | GGTTTTGAACGTCTCATTACCTGAT | 102 | *Enterococcus faecium* SF11770 | Peak et al. (2007) | 95 °C | 15 s |
|  | q_tetL-R | CCAATGGAAAAGGTTAACATAAAGG |  |  |  | 63 °C | 30 s |
|  | q_tetL-P | 6FAM-CCACCTGCGAGTACAAACTGGGTGAAC-TAMRA |  |  |  | 72 °C | 1 s |
| *tetS* | q_tetS-F | GTATGTTCATCTTTCTAAG | 190 | *Lactobacillus lactis* K214 | Manuzon et al., (2007) | 95 °C | 15 s |
|  | q_tetS-R | GCAATAACATCTTTTCAAC |  |  |  | 54 °C | 30 s |
|  | q_tetS-P | 6FAM-CCATGTGTCCAGGAGTATCTAC-TAMRA |  |  |  | 72 °C | 1 s |

Table S4. Composition of qPCR reactions with final concentrations of primers, probes, and buffer (LightCycler^®^ Probes Master, Roche Diagnostics Germany GmbH), as well as final volume of the template.

| qPCR component | Target gene | | | |
| --- | --- | --- | --- | --- |
|  | 16S rRNA | *aph(2’’)-Ia, aph(3’)-IIIa, ermA, ermB, sul1, tetL* | *sul2* | *tetS* |
| H_2_O, PCR-grade | ad. 20 µL | ad. 20 µL | ad. 20 µL | ad. 20 µL |
| Primer F | 0.8 µM | 0.5 µM | 0.25 µM | 0.75 µM |
| Primer R | 0.8 µM | 0.5 µM | 0.25 µM | 0.75 µM |
| Probe | 0.2 µM | 0.1 µM | 0.1 µM | 0.15 µM |
| Buffer | 1x | 1x | 1x | 1x |
| Template (1:100) | 5 µL | 5 µL | 5 µL | 5 µL |


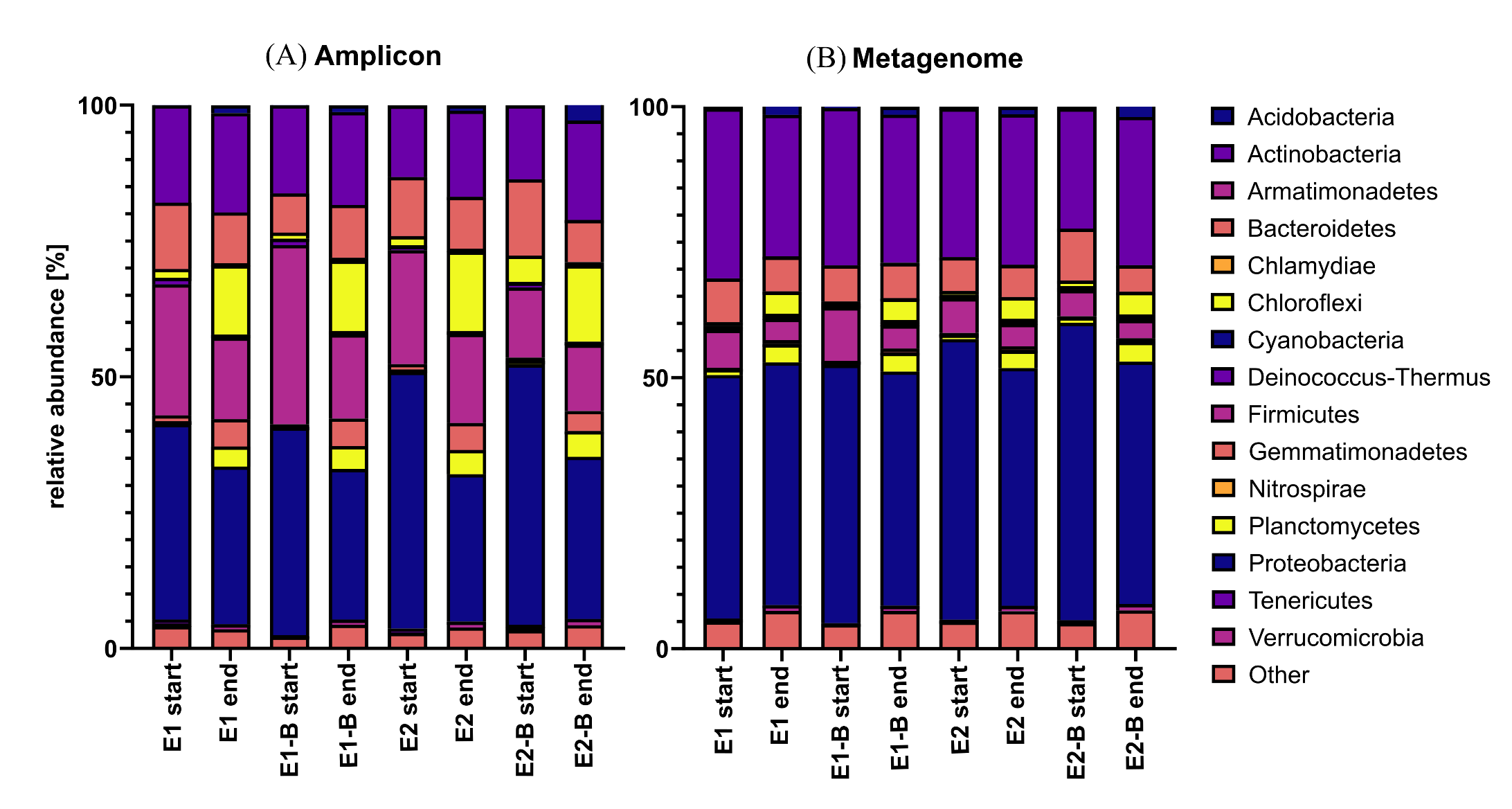


Figure S1. Relative abundance of bacterial phyla as assessed by 16S rRNA gene amplicon (A) and metagenomic sequencing (B). The composting trial comprised samples from start and end of composting of two repetitions (E1/E2), and two treatments (without and with biochar (B)), respectively. Amplicon sequencing was conducted on triplicate samples of mature compost. The average of these triplicates is depicted for better comparison with the metagenomic data. “Other” comprise unclassified OTUs, as well as phyla that were just present in either amplicon or metagenome data.


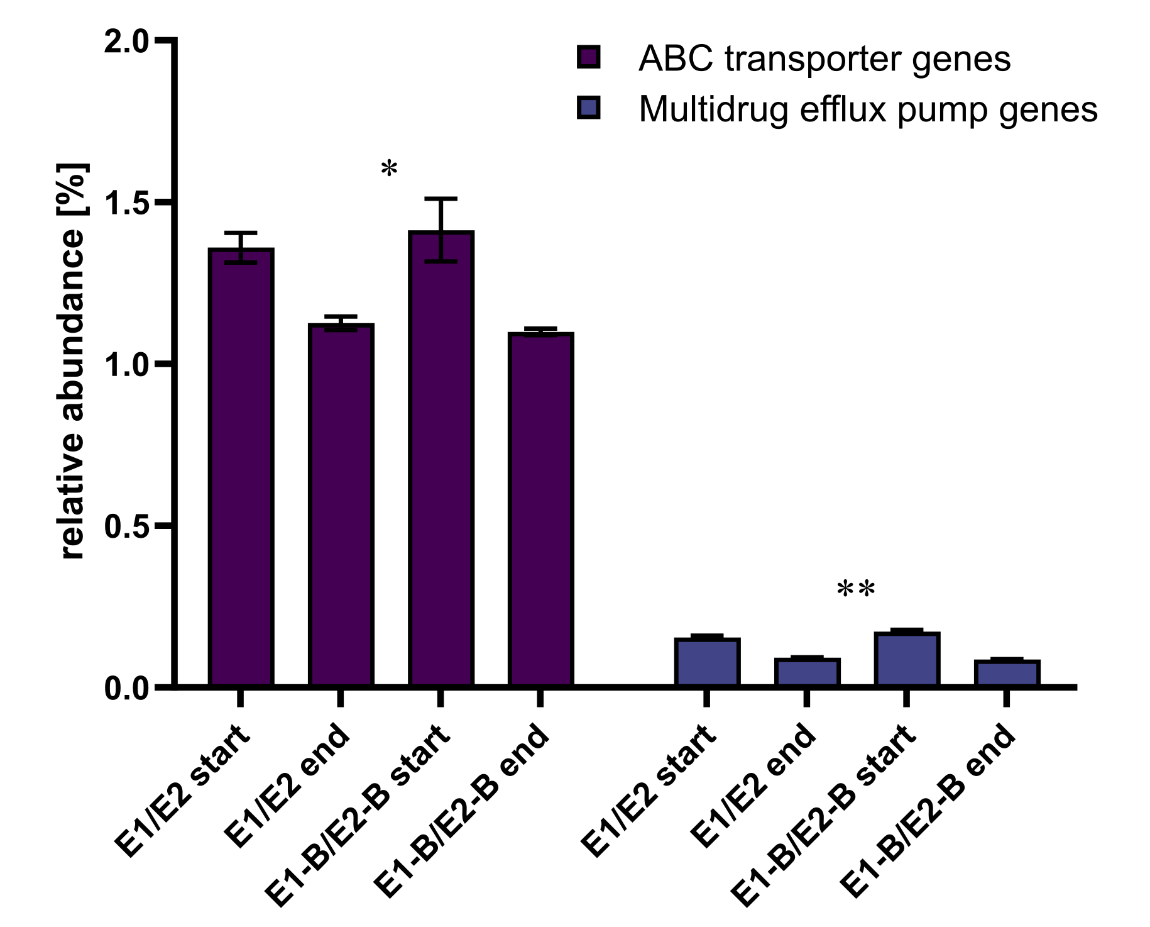


Figure S2. Relative abundance of ABC transporter and multidrug efflux pump genes as assigned by KEGG database. The bars depict the mean and standard deviation of the replicate compost piles without (E1/E2) and with biochar (E1-B/E2-B) for start and end of composting. 471 genes were assigned to ABC transporter and 27 to multidrug efflux pumps genes. Asterisks indicate the p-values obtained from Student’s t-test (** p < 0.01, * p < 0.05) representing the statistical significance of the data. P values were calculated to compare start with end samples of composting for all four compost piles combined (no differentiation of the treatments).

Table S5. qPCR results for aminoglycoside ARGs *aph(2’’)-Ia* and *aph(3‘)-IIIa* normalized to 1 g compost and 16S rRNA gene copies with standard deviation. Samples consist of two repetitions (E1 and E2), start, 14 day (14d) and end samples (triplicates: front (1), middle (2), back (3) of the compost piles). B indicates the biochar treatment of the compost trial.

| Compost Sample | *aph(2‘‘)-Ia* | | | | *aph(3‘)-IIIa* | | | |
| --- | --- | --- | --- | --- | --- | --- | --- | --- |
|  | Average Copy Number/ g compost | Standard deviation | Average Copies/  16S rRNA copies | Standard deviation | Average Copy Number/ g compost | Standard deviation | Average Copies/  16S rRNA copies | Standard deviation |
| E1 start | 1.27E+07 | 2.33E+06 | 0.000451 | 0.000091 | 2.68E+07 | 4.07E+06 | 0.001400 | 0.000558 |
| E1 14d | 2.62E+06 | 1.72E+05 | 0.000083 | 0.000011 | 1.46E+07 | 2.63E+06 | 0.000549 | 0.000181 |
| E1 end 1 | 1.06E+05 | 4.85E+04 | 0.000007 | 0.000003 | 2.36E+06 | 3.05E+05 | 0.000195 | 0.000081 |
| E1 end 2 | 1.49E+05 | 3.56E+04 | 0.000011 | 0.000003 | 2.21E+06 | 9.67E+05 | 0.000200 | 0.000105 |
| E1 end 3 | 9.46E+04 | 1.57E+04 | 0.000006 | 0.000001 | 1.42E+06 | 3.25E+05 | 0.000144 | 0.000059 |
| E1-B start | 8.60E+06 | 7.76E+05 | 0.000313 | 0.000032 | 3.63E+07 | 6.17E+06 | 0.001450 | 0.000584 |
| E1-B 14d | 9.48E+06 | 2.09E+06 | 0.000309 | 0.000082 | 3.22E+07 | 4.68E+06 | 0.001145 | 0.000429 |
| E1-B end 1 | 8.04E+04 | 1.33E+04 | 0.000005 | 0.000001 | 3.87E+06 | 7.40E+05 | 0.000339 | 0.000124 |
| E1-B end 2 | 9.42E+04 | 2.57E+04 | 0.000006 | 0.000002 | 2.79E+06 | 5.15E+05 | 0.000267 | 0.000105 |
| E1-B end 3 | 9.79E+04 | 3.99E+04 | 0.000006 | 0.000003 | 2.96E+06 | 6.15E+05 | 0.000285 | 0.000094 |
| E2 start | 1.61E+06 | 3.37E+05 | 0.000065 | 0.000014 | 2.20E+07 | 1.40E+06 | 0.000799 | 0.000313 |
| E2 end 1 | 5.79E+04 | 1.74E+04 | 0.000003 | 0.000001 | 2.07E+06 | 8.74E+05 | 0.000205 | 0.000116 |
| E2 end 2 | 6.41E+04 | 3.85E+04 | 0.000004 | 0.000003 | 2.17E+06 | 6.40E+05 | 0.000193 | 0.000092 |
| E2h end 3 | 5.75E+04 | 2.80E+04 | 0.000005 | 0.000003 | 2.39E+06 | 5.26E+05 | 0.000298 | 0.000117 |
| E2-B start | 7.58E+05 | 9.34E+04 | 0.000015 | 0.000003 | 6.86E+07 | 1.44E+07 | 0.002657 | 0.000934 |
| E2-B end 1 | 1.16E+05 | 3.23E+04 | 0.000010 | 0.000003 | 1.98E+06 | 4.16E+05 | 0.000257 | 0.000114 |
| E2-B end 2 | 1.32E+05 | 4.64E+04 | 0.000015 | 0.000006 | 1.49E+06 | 4.26E+05 | 0.000195 | 0.000088 |
| E2-B end 3 | 7.59E+04 | 3.75E+04 | 0.000004 | 0.000002 | 1.28E+06 | 3.47E+05 | 0.000146 | 0.000072 |

Table S6. qPCR results for erythromycin ARGs *ermA* and *ermB* normalized to 1 g compost and 16S rRNA gene copies with standard deviation. Samples consist of two repetitions (E1 and E2), start, 14 day (14d) and end samples (triplicates: front (1), middle (2), back (3) of the compost piles). B indicates the biochar treatment of the compost trial.

| Compost Sample | *ermA* | | | | *ermB* | | | |
| --- | --- | --- | --- | --- | --- | --- | --- | --- |
|  | Average Copy Number/ g compost | Standard deviation | Average Copies/  16S rRNA copies | Standard deviation | Average Copy Number/ g compost | Standard deviation | Average Copies/  16S rRNA copies | Standard deviation |
| E1 start | 4.18E+07 | 3.16E+07 | 0.001741 | 0.001394 | 2.18E+08 | 7.83E+07 | 0.011375 | 0.004087 |
| E1 14d | 2.42E+07 | 9.33E+06 | 0.001104 | 0.000502 | 3.89E+07 | 1.22E+07 | 0.001459 | 0.000458 |
| E1 end 1 | 2.45E+06 | 1.01E+06 | 0.000231 | 0.000099 | 5.31E+05 | 2.00E+05 | 0.000044 | 0.000017 |
| E1 end 2 | 3.09E+06 | 1.32E+06 | 0.000272 | 0.000129 | 5.20E+05 | 1.94E+05 | 0.000047 | 0.000018 |
| E1 end 3 | 2.02E+06 | 8.15E+05 | 0.000176 | 0.000077 | 5.58E+05 | 2.26E+05 | 0.000057 | 0.000023 |
| E1-B start | 1.22E+08 | 5.15E+07 | 0.005782 | 0.002947 | 5.19E+08 | 1.53E+08 | 0.020738 | 0.006101 |
| E1-B 14d | 3.93E+07 | 1.48E+07 | 0.001752 | 0.000724 | 2.29E+08 | 6.21E+07 | 0.008142 | 0.002209 |
| E1-B end 1 | 5.66E+06 | 2.92E+06 | 0.000474 | 0.000260 | 6.39E+05 | 2.78E+05 | 0.000056 | 0.000024 |
| E1-B end 2 | 4.01E+06 | 1.96E+06 | 0.000364 | 0.000187 | 4.00E+05 | 1.74E+05 | 0.000038 | 0.000017 |
| E1-B end 3 | 3.16E+06 | 1.33E+06 | 0.000281 | 0.000127 | 4.13E+05 | 1.66E+05 | 0.000040 | 0.000016 |
| E2 start | 3.33E+07 | 1.65E+07 | 0.001255 | 0.000735 | 1.42E+08 | 4.13E+07 | 0.005156 | 0.001502 |
| E2 end 1 | 2.33E+06 | 9.43E+05 | 0.000192 | 0.000082 | 3.93E+05 | 1.37E+05 | 0.000039 | 0.000014 |
| E2 end 2 | 2.29E+06 | 1.26E+06 | 0.000232 | 0.000138 | 3.16E+05 | 1.10E+05 | 0.000028 | 0.000010 |
| E2h end 3 | 3.23E+06 | 1.65E+06 | 0.000403 | 0.000212 | 4.19E+05 | 1.63E+05 | 0.000052 | 0.000020 |
| E2-B start | 1.55E+07 | 7.23E+06 | 0.000445 | 0.000229 | 2.31E+07 | 7.72E+06 | 0.000893 | 0.000299 |
| E2-B end 1 | 3.31E+06 | 1.70E+06 | 0.000472 | 0.000256 | 2.88E+05 | 9.29E+04 | 0.000037 | 0.000012 |
| E2-B end 2 | 2.84E+06 | 1.47E+06 | 0.000366 | 0.000202 | 1.21E+05 | 9.70E+04 | 0.000016 | 0.000013 |
| E2-B end 3 | 2.82E+06 | 1.36E+06 | 0.000248 | 0.000131 | 3.34E+05 | 6.92E+04 | 0.000038 | 0.000008 |

Table S7. qPCR results for sulfonamide ARGs *sul1* and *sul2* normalized to 1 g compost and 16S rRNA gene copies with standard deviation. Samples consist of two repetitions (E1 and E2), start, 14 day (14d) and end samples (triplicates: front (1), middle (2), back (3) of the compost piles). B indicates the biochar treatment of the compost trial.

| Compost Sample | *sul1* | | | | *sul2* | | | |
| --- | --- | --- | --- | --- | --- | --- | --- | --- |
|  | Average Copy Number/ g compost | Standard deviation | Average Copies/  16S rRNA copies | Standard deviation | Average Copy Number/ g compost | Standard deviation | Average Copies/  16S rRNA copies | Standard deviation |
| E1 start | 1.88E+09 | 6.99E+08 | 0.098288 | 0.051408 | 1.17E+09 | 1.48E+08 | 0.060916 | 0.023733 |
| E1 14d | 1.41E+09 | 5.94E+08 | 0.052969 | 0.026670 | 4.62E+08 | 4.01E+07 | 0.017306 | 0.005020 |
| E1 end 1 | 1.81E+08 | 7.68E+07 | 0.014948 | 0.008651 | 2.80E+07 | 3.36E+06 | 0.002315 | 0.000953 |
| E1 end 2 | 1.55E+08 | 6.54E+07 | 0.014013 | 0.007212 | 2.48E+07 | 2.22E+06 | 0.002245 | 0.000689 |
| E1 end 3 | 7.93E+07 | 3.33E+07 | 0.008038 | 0.004334 | 2.03E+07 | 2.62E+06 | 0.002053 | 0.000743 |
| E1-B start | 2.48E+09 | 8.30E+08 | 0.098996 | 0.049022 | 1.29E+09 | 1.41E+08 | 0.051600 | 0.019652 |
| E1-B 14d | 2.37E+09 | 7.54E+08 | 0.084173 | 0.039519 | 8.31E+08 | 1.57E+08 | 0.029550 | 0.011618 |
| E1-B end 1 | 1.73E+08 | 7.02E+07 | 0.015147 | 0.007746 | 3.36E+07 | 3.75E+06 | 0.002941 | 0.000972 |
| E1-B end 2 | 1.54E+08 | 6.40E+07 | 0.014696 | 0.007947 | 2.95E+07 | 4.25E+06 | 0.002821 | 0.001056 |
| E1-B end 3 | 1.39E+08 | 5.44E+07 | 0.013379 | 0.006253 | 2.95E+07 | 4.21E+06 | 0.002842 | 0.000833 |
| E2 start | 2.09E+09 | 7.52E+08 | 0.075974 | 0.040109 | 6.91E+08 | 8.50E+07 | 0.025100 | 0.010186 |
| E2 end 1 | 8.44E+07 | 3.45E+07 | 0.008347 | 0.004656 | 2.30E+07 | 3.03E+06 | 0.002271 | 0.000913 |
| E2 end 2 | 8.52E+07 | 3.97E+07 | 0.007565 | 0.004530 | 2.37E+07 | 2.52E+06 | 0.002108 | 0.000823 |
| E2h end 3 | 1.22E+08 | 5.19E+07 | 0.015177 | 0.008142 | 2.15E+07 | 1.90E+06 | 0.002679 | 0.000901 |
| E2-B start | 1.47E+09 | 6.13E+08 | 0.056992 | 0.028674 | 6.91E+08 | 9.41E+07 | 0.026774 | 0.008373 |
| E2-B end 1 | 8.57E+07 | 3.19E+07 | 0.012939 | 0.007036 | 1.79E+07 | 1.76E+06 | 0.002312 | 0.000930 |
| E2-B end 2 | 9.50E+07 | 4.17E+07 | 0.012398 | 0.006987 | 1.86E+07 | 2.65E+06 | 0.002422 | 0.000922 |
| E2-B end 3 | 1.20E+08 | 6.15E+07 | 0.013614 | 0.008984 | 1.97E+07 | 1.78E+06 | 0.002246 | 0.000949 |

Table S8. qPCR results for tetracycline ARGs *tetL* and *tetS* normalized to 1 g compost and 16S rRNA gene copies with standard deviation. Samples consist of two repetitions (E1 and E2), start, 14 day (14d) and end samples (triplicates: front (f), middle (m), back (b) of the compost piles). B indicates the biochar treatment of the compost trial.

| Compost Sample | *tetL* | | | | *tetS* | | | |
| --- | --- | --- | --- | --- | --- | --- | --- | --- |
|  | Average Copy Number/ g compost | Standard deviation | Average Copies/  16S rRNA copies | Standard deviation | Average Copy Number/ g compost | Standard deviation | Average Copies/  16S rRNA copies | Standard deviation |
| E1 start | 9.77E+08 | 7.22E+08 | 0.039442 | 0.025995 | 5.11E+06 | 4.70E+05 | 0.000184 | 0.000018 |
| E1 14d | 4.08E+08 | 2.56E+08 | 0.015806 | 0.011225 | 8.64E+05 | 1.13E+05 | 0.000028 | 0.000004 |
| E1 end 1 | 3.41E+07 | 2.29E+07 | 0.002917 | 0.002329 | 1.34E+04 | 1.29E+04 | 0.000001 | 0.000001 |
| E1 end 2 | 3.98E+07 | 2.77E+07 | 0.003680 | 0.002829 | 1.32E+04 | 9.90E+03 | 0.000001 | 0.000001 |
| E1 end 3 | 3.73E+07 | 2.88E+07 | 0.003633 | 0.002978 | 2.47E+04 | 2.39E+04 | 0.000002 | 0.000002 |
| E1-B start | 1.74E+09 | 1.12E+09 | 0.071849 | 0.054641 | 4.60E+06 | 3.51E+05 | 0.000168 | 0.000015 |
| E1-B 14d | 1.10E+09 | 7.30E+08 | 0.038108 | 0.027961 | 1.38E+06 | 7.33E+04 | 0.000045 | 0.000007 |
| E1-B end 1 | 6.07E+07 | 4.65E+07 | 0.005280 | 0.004345 | 4.53E+04 | 5.58E+04 | 0.000003 | 0.000003 |
| E1-B end 2 | 5.36E+07 | 3.46E+07 | 0.005221 | 0.003894 | 2.94E+04 | 3.02E+04 | 0.000002 | 0.000002 |
| E1-B end 3 | 5.80E+07 | 3.74E+07 | 0.005741 | 0.004098 | 5.42E+03 | 5.36E+03 | 0.000000 | 0.000000 |
| E2 start | 5.26E+08 | 3.41E+08 | 0.018933 | 0.014203 | 5.16E+05 | 1.05E+05 | 0.000021 | 0.000004 |
| E2 end 1 | 5.25E+07 | 4.32E+07 | 0.004886 | 0.004262 | 3.23E+04 | 1.12E+04 | 0.000002 | 0.000001 |
| E2 end 2 | 3.79E+07 | 2.46E+07 | 0.003397 | 0.002567 | 2.76E+04 | 3.13E+04 | 0.000002 | 0.000002 |
| E2h end 3 | 3.50E+07 | 2.47E+07 | 0.004388 | 0.003421 | 1.40E+04 | 1.97E+04 | 0.000001 | 0.000002 |
| E2-B start | 1.12E+08 | 7.85E+07 | 0.004340 | 0.003299 | 9.07E+05 | 2.09E+05 | 0.000018 | 0.000005 |
| E2-B end 1 | 2.88E+07 | 2.07E+07 | 0.003985 | 0.003413 | 3.67E+04 | 4.61E+04 | 0.000003 | 0.000004 |
| E2-B end 2 | 2.57E+07 | 1.92E+07 | 0.003254 | 0.002634 | 1.81E+04 | 1.16E+04 | 0.000002 | 0.000001 |
| E2-B end 3 | 2.41E+07 | 1.74E+07 | 0.002740 | 0.002274 | 2.76E+04 | 3.90E+04 | 0.000001 | 0.000002 |

References

Böckelmann, U., Dörries, H. H., Ayuso-Gabella, M. N., De Marçay, M. S., Tandoi, V., Levantesi, C., Masciopinto, C., Houtte, E. Van, Szewzyk, U., Wintgens, T., & Grohmann, E. (2009). Quantitative PCR monitoring of antibiotic resistance genes and bacterial pathogens in three european artificial groundwater recharge systems. *Applied and Environmental Microbiology*, *75*(1), 154–163. https://doi.org/10.1128/AEM.01649-08

Heuer, H., Focks, A., Lamshöft, M., Smalla, K., Matthies, M., & Spiteller, M. (2008). Fate of sulfadiazine administered to pigs and its quantitative effect on the dynamics of bacterial resistance genes in manure and manured soil. *Soil Biology and Biochemistry*, *40*(7), 1892–1900. https://doi.org/10.1016/j.soilbio.2008.03.014

Heuer, H., & Smalla, K. (2007). Manure and sulfadiazine synergistically increased bacterial antibiotic resistance in soil over at least two months. *Environmental Microbiology*, *9*(3), 657–666. https://doi.org/10.1111/j.1462-2920.2006.01185.x

Manuzon, M. Y., Hanna, S. E., Luo, H., Yu, Z., Harper, W. J., & Wang, H. H. (2007). Quantitative assessment of the tetracycline resistance gene pool in cheese samples by real-time TaqMan PCR. *Applied and Environmental Microbiology*, *73*(5), 1676–1677. https://doi.org/10.1128/AEM.01994-06

Peak, N., Knapp, C. W., Yang, R. K., Hanfelt, M. M., Smith, M. S., Aga, D. S., & Graham, D. W. (2007). Abundance of six tetracycline resistance genes in wastewater lagoons at cattle feedlots with different antibiotic use strategies. *Environmental Microbiology*, *9*(1), 143–151. https://doi.org/10.1111/j.1462-2920.2006.01123.x

Takai, K., & Horikoshi, K. (2000). Rapid detection and quantification of members of the archaeal community by quantitative PCR using fluorogenic probes. *Applied and Environmental Microbiology*, *66*(11), 5066–5072. https://doi.org/10.1128/AEM.66.11.5066-5072.2000

Woegerbauer, M., Zeinzinger, J., Springer, B., Hufnagl, P., Indra, A., Korschineck, I., Hofrichter, J., Kopacka, I., Fuchs, R., Steinwider, J., Fuchs, K., Nielsen, K. M., & Allerberger, F. (2014). Prevalence of the aminoglycoside phosphotransferase genes *aph(3’)-IIIa and aph(3’)-IIa* in *Escherichia coli*, *Enterococcus faecalis*, *Enterococcus faecium*, *Pseudomonas aeruginosa*, *Salmonella enterica* subsp. Enterica and *Staphylococcus aureus* isolates in Austria. *Journal of Medical Microbiology*, *63*, 210–217. https://doi.org/10.1099/jmm.0.065789-0
